# Natural source, bioactivity and synthesis of benzofuran derivatives

**DOI:** 10.1039/c9ra04917g

**Published:** 2019-09-02

**Authors:** Yu-hang Miao, Yu-heng Hu, Jie Yang, Teng Liu, Jie Sun, Xiao-jing Wang

**Affiliations:** School of Medicine and Life Sciences, University of Jinan, Shandong Academy of Medical Sciences Jinan 250200 Shandong China 931077311@qq.com 871534141@qq.com 1172493743@qq.com liuteng823@163.com sunjie310@126.com xiaojing6@gmail.com; Institute of Materia Medica, Shandong Academy of Medical Sciences Jinan 250062 Shandong China; Key Laboratory for Biotech-Drugs Ministry of Health Jinan 250062 Shandong China; Key Laboratory for Rare & Uncommon Diseases of Shandong Province Jinan 250062 Shandong China

## Abstract

Benzofuran compounds are a class of compounds that are ubiquitous in nature. Numerous studies have shown that most benzofuran compounds have strong biological activities such as anti-tumor, antibacterial, anti-oxidative, and anti-viral activities. Owing to these biological activities and potential applications in many aspects, benzofuran compounds have attracted more and more attention of chemical and pharmaceutical researchers worldwide, making these substances potential natural drug lead compounds. For example, the recently discovered novel macrocyclic benzofuran compound has anti-hepatitis C virus activity and is expected to be an effective therapeutic drug for hepatitis C disease; novel scaffold compounds of benzothiophene and benzofuran have been developed and utilized as anticancer agents. Novel methods for constructing benzofuran rings have been discovered in recent years. A complex benzofuran derivative is constructed by a unique free radical cyclization cascade, which is an excellent method for the synthesis of a series of difficult-to-prepare polycyclic benzofuran compounds. Another benzofuran ring constructed by proton quantum tunneling has not only fewer side reactions, but also high yield, which is conducive to the construction of complex benzofuran ring systems. This review summarizes the recent studies on the various aspects of benzofuran derivatives including their important natural product sources, biological activities and drug prospects, and chemical synthesis, as well as the relationship between the bioactivities and structures.

## Abstract 2

Benzofuran compounds are a class of compounds that are ubiquitous in nature.

## Graphical abstract

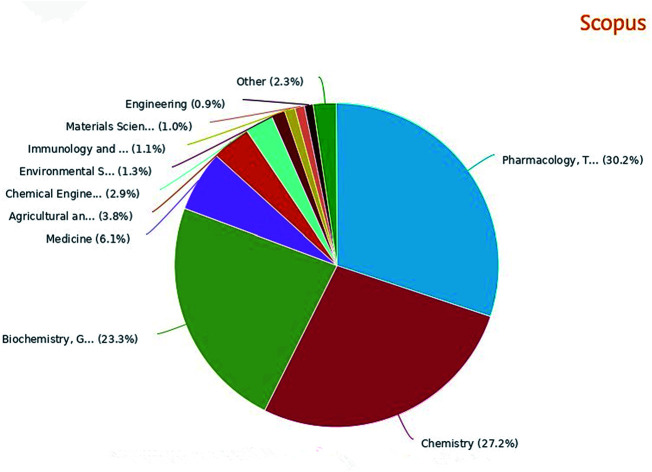

## 1. Introduction

**Fig. 1:**
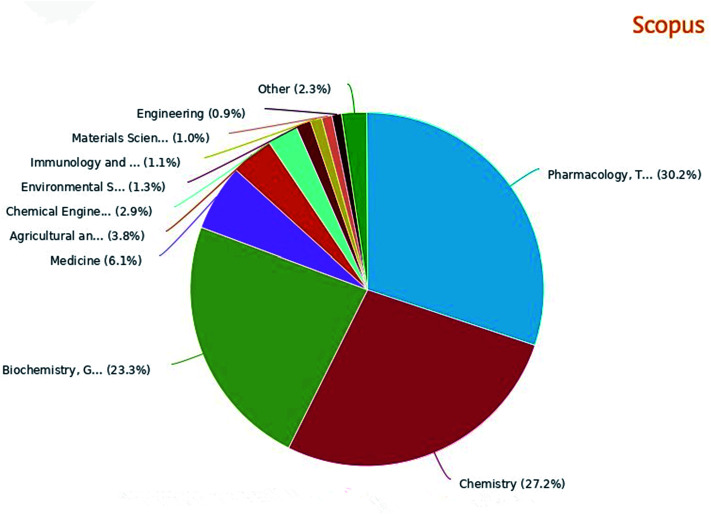
Literature of benzofuran compounds by subject category in the past decade.

**Fig. 2:**
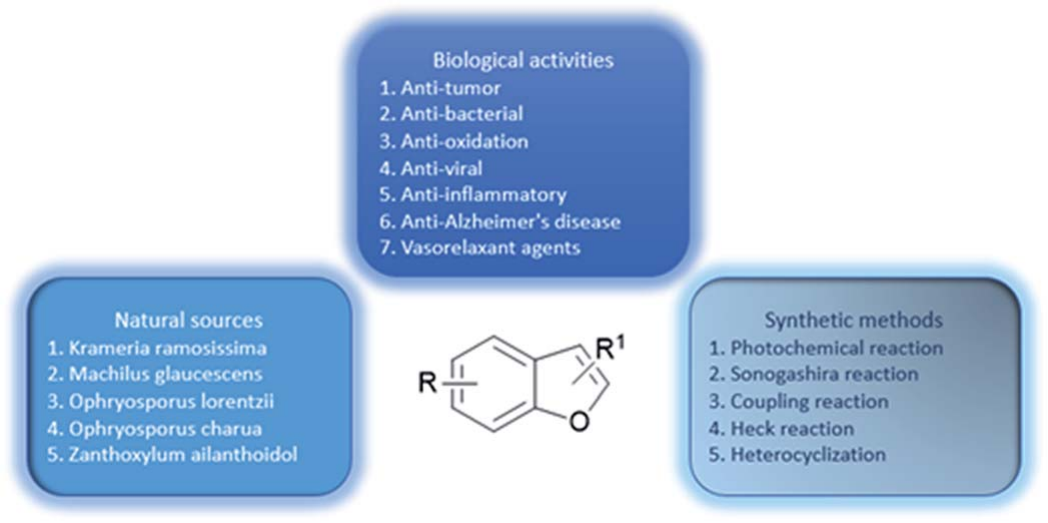
Comprehensive understanding of benzofurans through biological activities, natural sources, and synthetic methods.

A great number of heterocyclic compounds and heterocyclic fragments are present in many drugs due to their versatility and unique physicochemical properties and have become an important basis for medicinal chemistry.^1^ Many significant natural products and natural medicines have these structures. Natural products containing benzofuran rings are the main source of some drugs and clinical drug candidates.^2^ The heterocyclic compound having a benzofuran ring as a core is a basic structural unit of various biologically active natural medicines and synthetic chemical raw materials.^3^ The broad range of clinical uses of benzofuran derivatives indicate the diverse pharmacological activities of this series of compounds, so benzofuran and its derivatives have attracted much attention owing to their biological activities (Fig. 1 percentage distribution of various subject categories) and potential applications as drugs.^4^ Benzofuran compounds are widely distributed in higher plants such as Asteraceae, Rutaceae, Liliaceae, and Cyperaceae. The number of such compounds discovered from Asteraceae is the highest.^5^ Studies have found that benzofuran and its derivatives are diverse in nature and exist widely in natural and non-natural compounds. The natural products containing benzofuran compounds are mainly isolated from *Krameria ramosissima*, *Machilus glaucescens*, *Ophryosporus lorentzii*, *Ophryosporus charua* and *Zanthoxylum ailanthoidol*. These compounds have a wide range of biological and pharmacological activities, and therefore have great values in the field of new drug research.^6,7^ Moreover, benzofuran derivatives are also biodynamic agents that can be used to design and develop new potential therapeutic agents.^8^ In recent years, researchers have found that such compounds have various biological activities including: anti-tumor,^9^ antibacterial,^10,11^ anti-oxidative,^1,12,13^ anti-AD,^14^ anti-parasitic,^15^ anti-acetylcholine,^16^ and anti-inflammatory activities.^17^ They can also be used as bone anabolic agent,^18^ and fluorescent sensor for analgesic^19^ The most well-known and recognized natural products containing benzofuran ring structure, include ailanthoidol, amiodarone and bufuralol compounds.^20,21^ In addition, some 2-arylbenzofurans derived from natural products also have good biological activities,^22^ such as anti-cancer,^23^ anti-inflammatory,^24^ anti-oxidative,^25^ and antibacterial activities^26,27^ Recently, an oral active and blood–brain barrier permeable benzofuran analog has been found to exhibit potent anti-amyloid aggregation activity, which can provide an alternative treatment for Alzheimer's disease (AD).^28^ In addition, the benzofuran analog oxazolidine was found to be a potential multifunctional molecule, and its anti-proliferative activity allows it to plays an important role in the treatment of tumors.^29^ Benzofuran compounds are expected to be important compounds for the treatment of multifactorial diseases. This review includes five parts: the drugs on the market with benzofuran structure, (1) (2) the main natural sources, (3) diversified pharmacological activities, (4) common synthetic methods and (5) synthetic examples of various active compounds (a summary of the review is shown in Fig. 2). The purpose of this review is to summarize the recently reported natural sources of benzofuran derivatives, the research progress of their biological activities and the synthesis of some common benzofuran compounds, to help readers deeply understand the important roles of benzofurans in medicinal chemistry.

## 2Examples of drugs containing a benzofuran moiety

The most recognized benzofuran compounds with extensive pharmaceutical applications include amiodarone, angelicin, bergapten, nodekenetin, xanthotoxin, and usnic acid. These compounds have been widely used in antiarrhythmic, dermatological and anticancer therapy, illustrating the critical clinical application value of benzofuran compounds and the significant potentials for these compounds in drug development in the future.

**Fig. 3:**
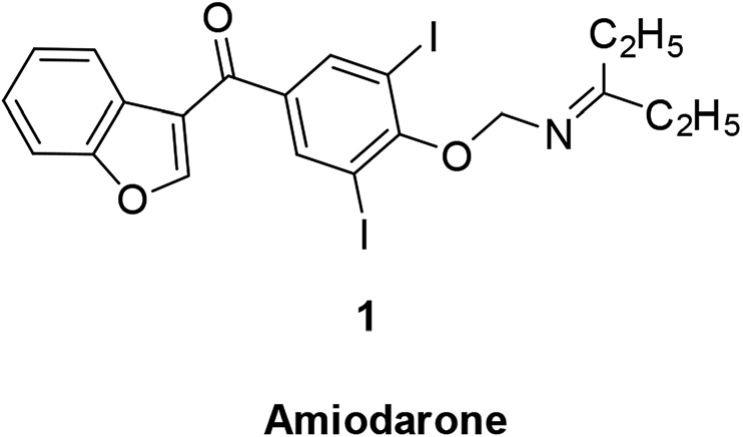
Structure of benzofuran derivative amiodarone.

The broad-spectrum antiarrhythmic drug amiodarone is a representative benzofuran drug. The drug can inhibit rapid sodium ion influx in atrial and myocardial conduction fibers, slow down conduction velocity, reduce sinus node autonomy, and has a good effect in dealing with paroxysmal supraventricular tachycardia, atrial premature beats, and premature ventricular contractions. It is mainly used as an antiarrhythmic and anti-angina drug (Fig. 3).^30,31^

**Fig. 4:**
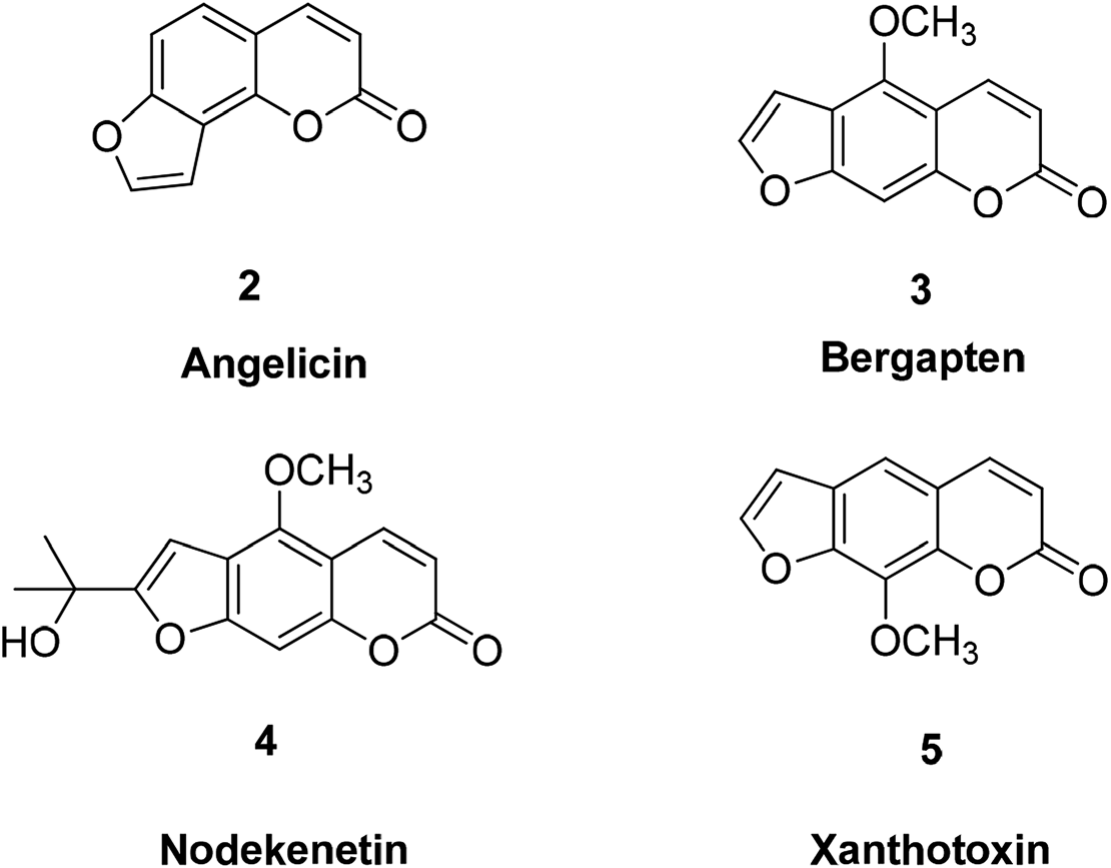
Structure of benzofuran derivative psoralen.

Psoralen is a furocoumarin compound formed by fusion of furan with coumarin, which is naturally exists in some plant species or is synthesized *in vitro* (Fig. 4). Psoralen exists in many natural plants, including limes, lemons and parsnips. An important feature of furocoumarin compounds is their ability to produce singlet oxygen.^32^

It has been reported that the furocoumarin angelicin (2) found in the fruit of *Psoralea corylifolia* L. is structurally related to psoralen, a well-known chemical photosensitizer, which is used for treatment of different skin diseases such as psoriasis and vitiligo due to its anti-proliferative effect.^33,34^ Anti-cancer studies treatment have found that angelicin is a natural compound that effectively induces apoptosis in human SH-SY5Y neuroblastoma cells, indicating this compound has potential effect in treatment of human neuroblastoma cancer.^35^

Bergapten (3) is a conventional photochemotherapy drug for psoriasis^36^ in the course of cancer treatment. Bergapten can be combined with other forms of targeted chemotherapy to improve cancer treatment outcomes based on its metabolic targeting.^37^

In anti-inflammatory treatment, bergapten can participate in the treatment of inflammation by inhibiting the production of pro-inflammatory cytokines.^38^

Nodekenetin (4) and xanthotoxin (5) are effective against skin diseases including cutaneous T-cell lymphoma, vitiligo, atopic dermatitis, and psoriasis.^32^

**Fig. 5:**
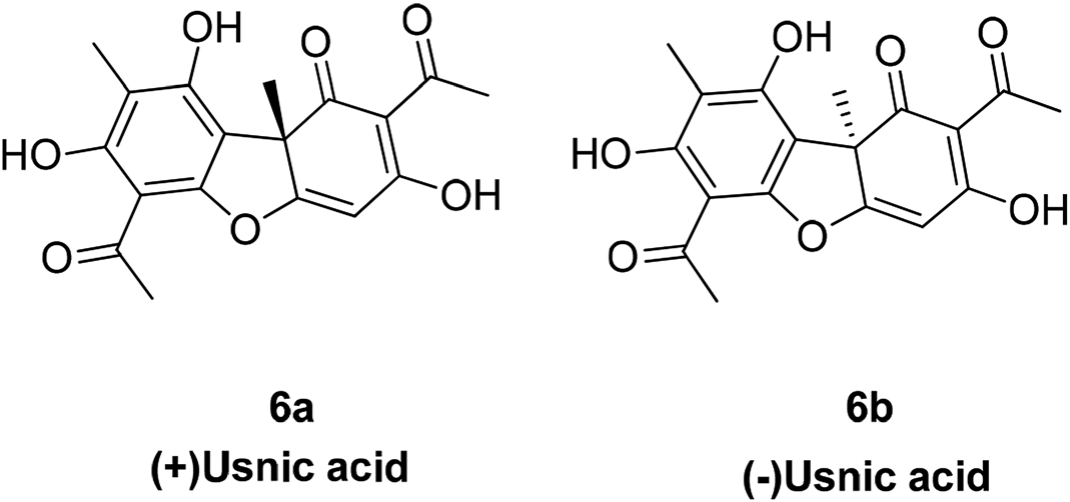
Structure of compound usnic acid.

Usnic acid is an antibiotic and both its (+) and (−) enantiomers (Fig. 5) are effective against a variety of Gram-positive (G+) bacterial strains; notably, they can inhibit the growth of multi-drug resistant strains such as *S. aureus*, *Enterococcus* and *Mycobacterium*. The (+)-usnic acid appears to be selective for *Streptococcus mutans* without effects on the oral saprophytic flora.^39^ Usnic acid also has potential anti-tumor activity as well as other biological properties against mitosis and antioxidants.^40–42^

## 3. Natural compounds containing benzofuran ring

Benzofuran compounds are a class of organic compounds widely distributed in nature and have long been the focus of attention. Thousands of natural compounds have been discovered and isolated so far. This part will review the benzofuran compounds discovered and isolated from natural animals and plants in recent years (Table 1).

**Table 1:** Natural products have been obtained in recent years from biologically active compounds and benzofuran compounds

Structure	Genus and species name	Territorial	Extraction isolation year	Biological activity		Ref.
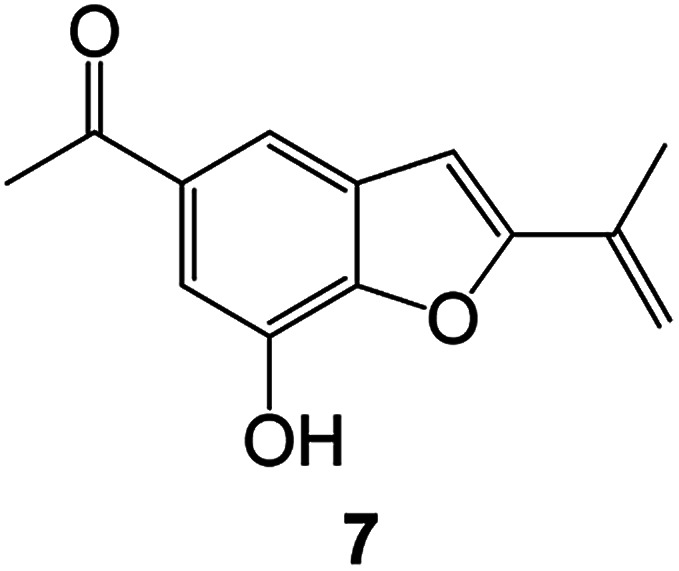	(Asteraceae) *Ageratina adenophora*	Mexico	2018	Anti-fungal activity	Dehydrotrienone benzofuran derivative, eco-friendly antifungal agent	43
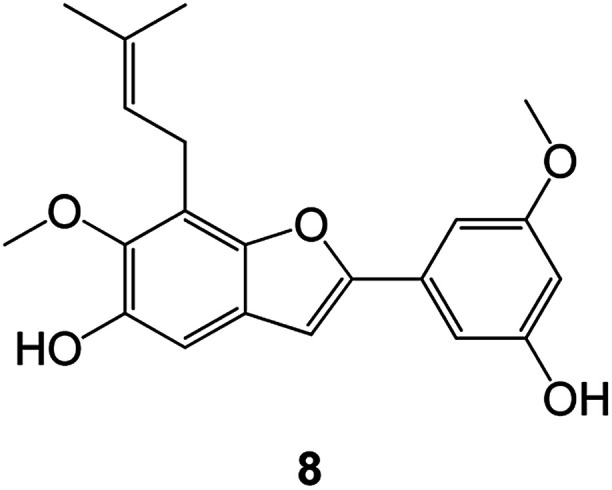	(Fabaceae) *Calpocalyx dinklagei*	Western Central Africa	2017	Anti-inflammatory	Inflammatory disease multi-target agent	44
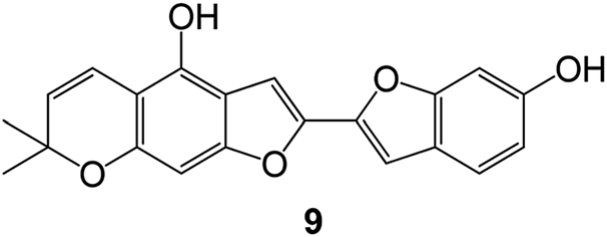	(Artocarpus) *Artocarpus heterophyllus*	Tropical regions of Asia	2017	Anti-cancer activity	Cytotoxic activity against human oral cancer (KB), human breast cancer (MCF-7) and lung cancer (NCI-H187) cell lines	45
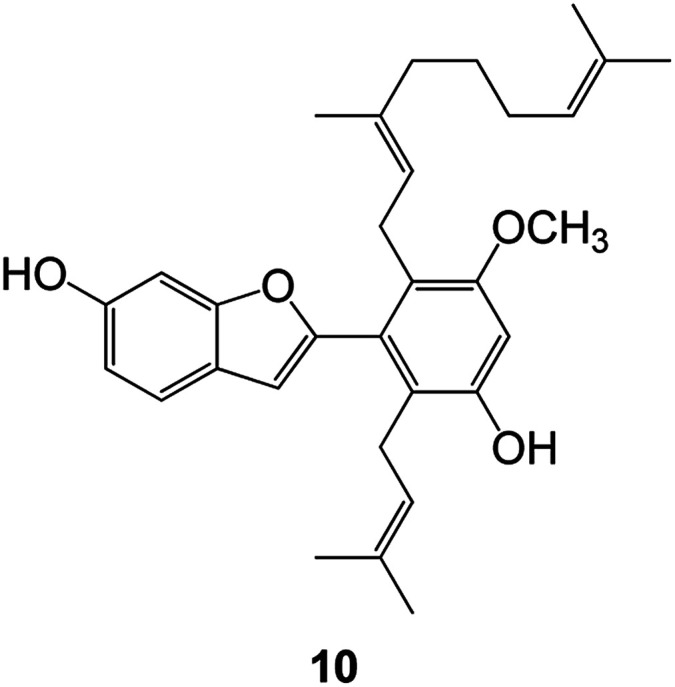	(Moraceae) *Artocarpus lakoocha*	Asia and Southeast Asia	2017	AChE and BChE inhibitory	As a potential new anti-ChE agent	46
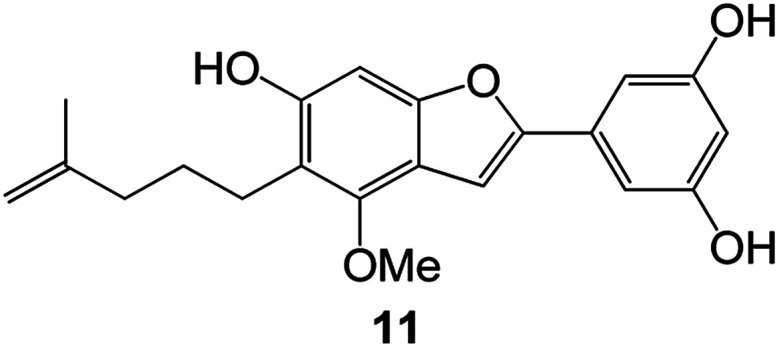	(Moraceae) *Chlorophora regia*	Tropical West Africa, Senegal, Gambia and Ghana	2016	Anti-inflammatory	As an antioxidant inhibitor	25
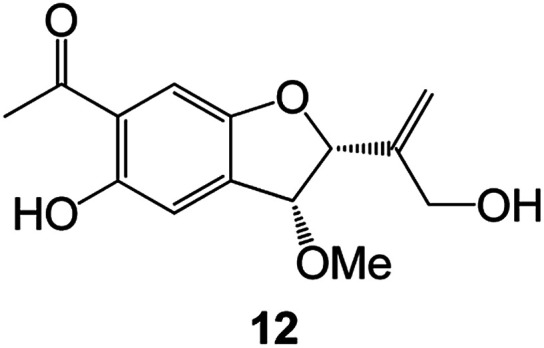	(*Asterothamnu*) *Asterothamnus centrali-asiaticus*	Gansu, Nei Mongol, Ningxia, Qinghai, SE Xinjiang (S Mongolia)	2016	Anti-oxidant	As a potential antioxidant	47
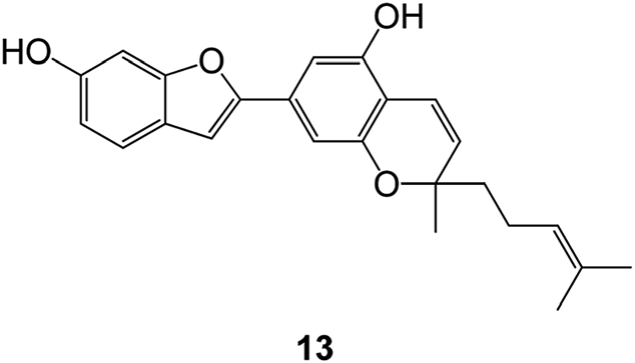	(Moraceae) *Morus alba*	Asia (Vietnam, China, Japan, and South Korea)	2016	Inhibition of pancreatic lipase	Effectively inhibit pancreatic lipase as a potential diet pills	48
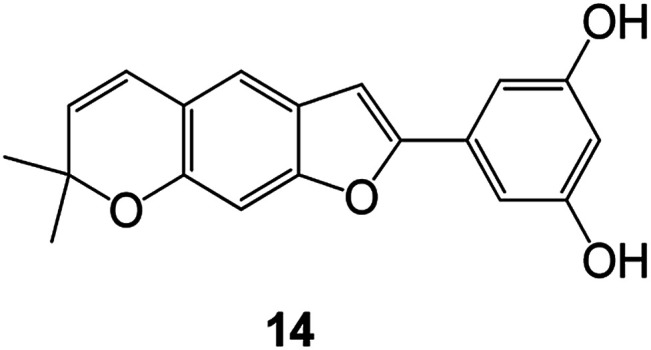	(Moraceae) *Morus nigra*	West Asia	2018	Anti-tumor	Multifunctional anti-tumor agent	49
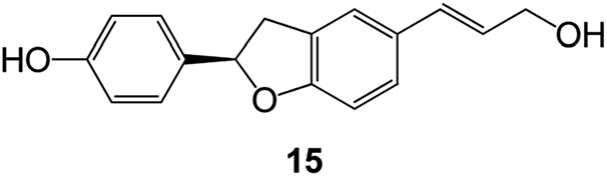	(Butterbure) *Mappianthus iodoies*	Southern China	2017	Anti-cancer activity	Cytotoxicity against HL-60, SMMC7721, A-549, MCF-7 and SW-480	50
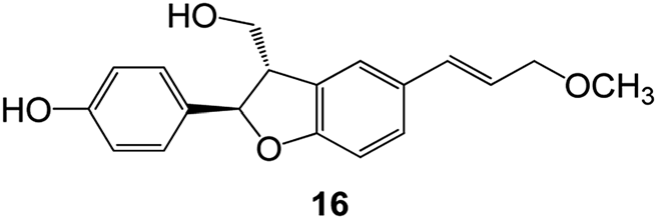						
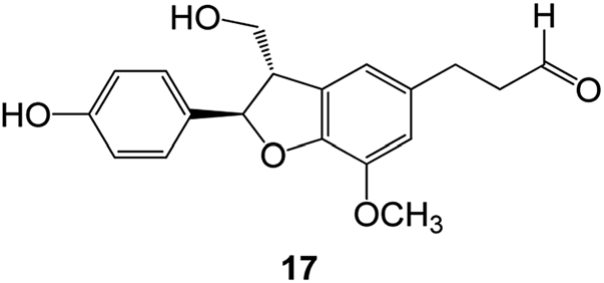						
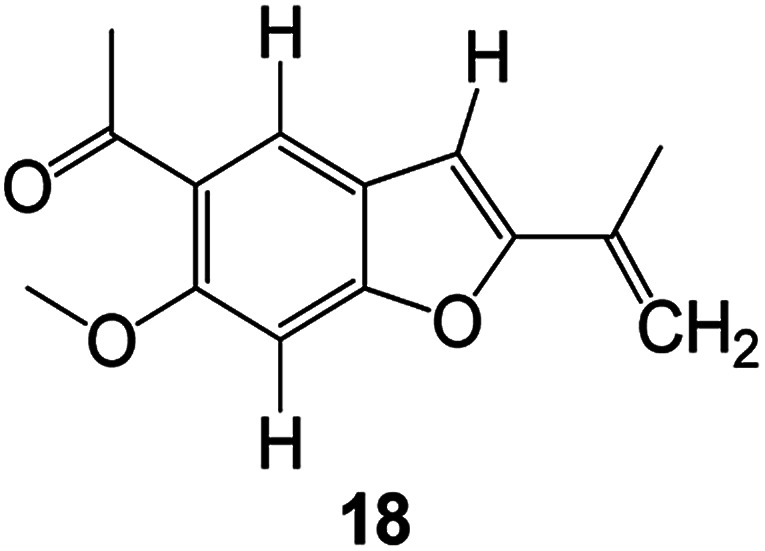	(Butterbure) *Petasites hybridus*	Europe, West Asia, North America	2015	Anti-cancer activity	It has cytotoxic and apoptotic effects on human breast cancer MCF-7 cells	51
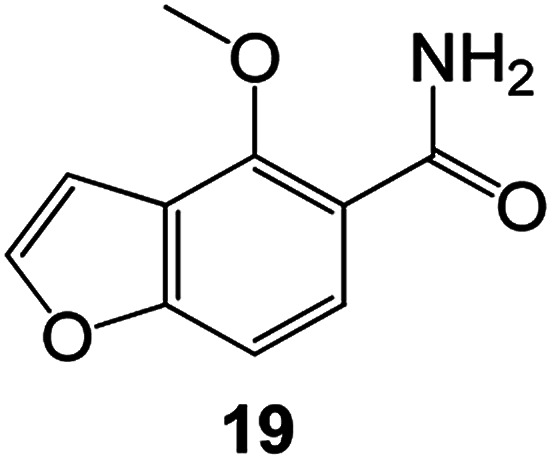	(Fabaceae) *Tephrosia purpurea*	Eastern India to Central Bangladesh	2015	Anti-allergic activity	For the treatment of allergic diseases, including rhinitis	52
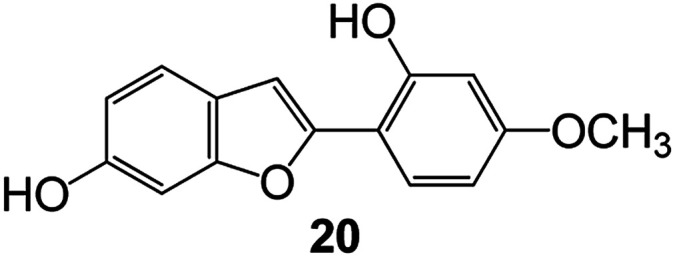	(Leguminosae) *Sophora tonkinensis*	South China, Korea	2014	Anti-allergic activity	Inhibition of IL-6 production in HMC-1 cells produces anti-allergic effects	53
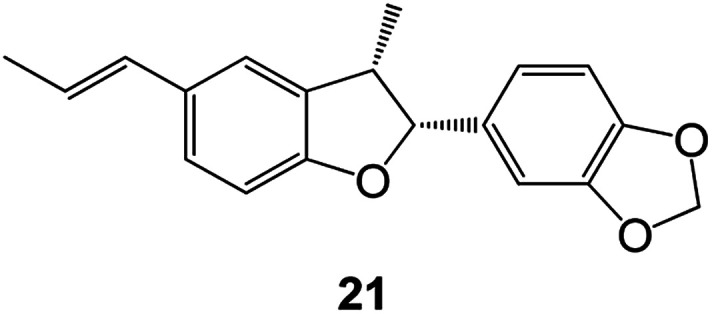	(Aristolochiaceae) *Aristolochia fordiana*	Southwestern China	2013	Anti-oxidation activity	Inhibition of NO release in cells	54
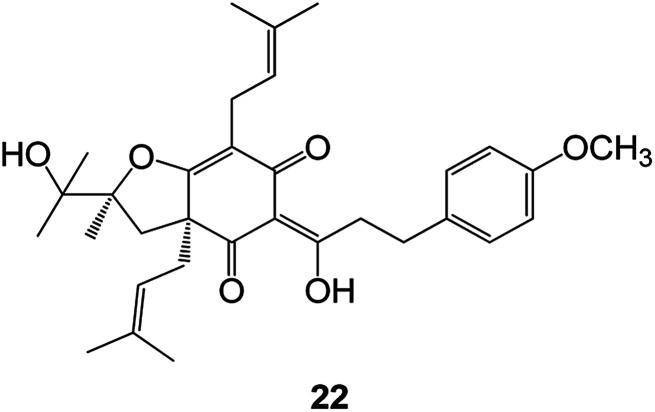	*Flemingia philippinensis*	Southern Asia	2012	Anti-oxidation activity	Potential antioxidant	55
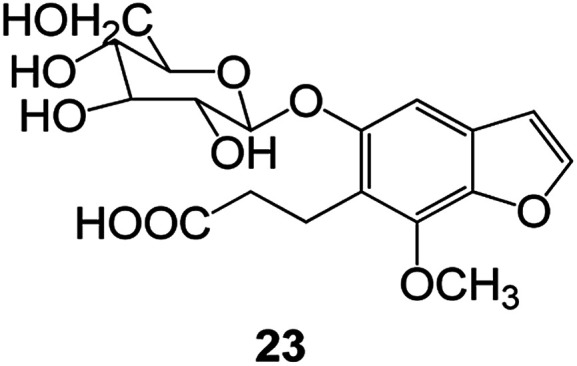	(Ficus) *Ficus tikoua* Bur	South China, India, Vietam and Laos	2011	Anti-oxidation activity	As a source of antioxidants	56
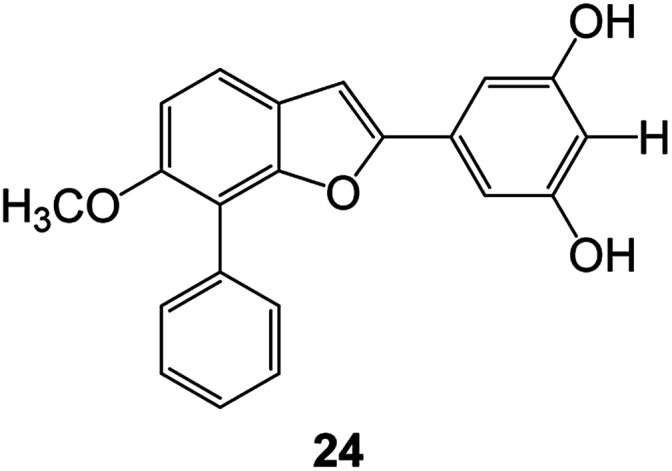	(Moraceae) *Morusalba* var. *multicaulis*	Throughout Asia, Europe, North and South America, and Africa	2011	Anti-obesity and anti-inflammatory activity	Potential drug with anti-obesity and anti-inflammatory activities	57
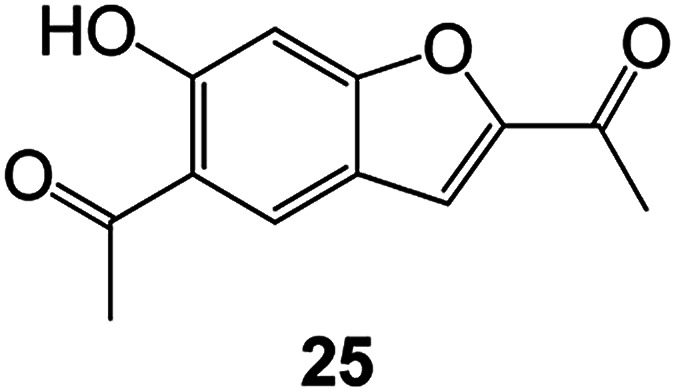	Radix Eupatorii Chinensis	Southern China	2009	Anti-fungal, anti-oxidation and anti-tumor activity	Potential multi-targeted drug with antifungal, anti-oxidant and anti-tumor activities	58
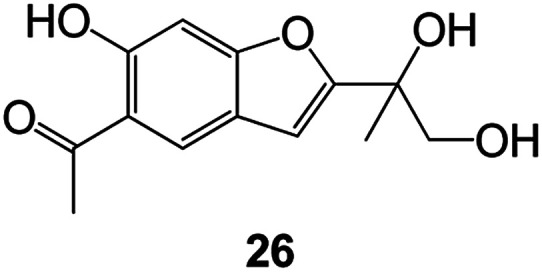						
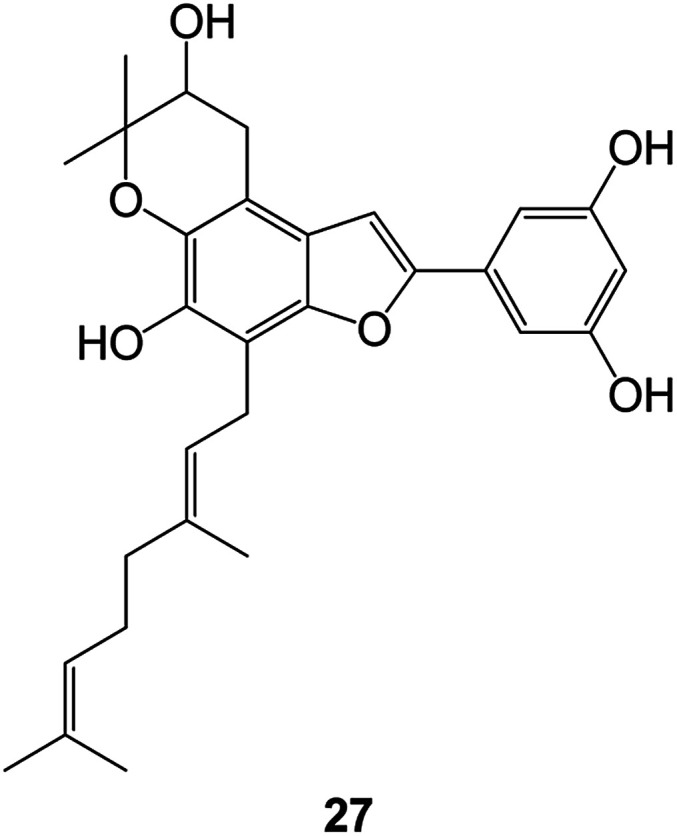	(*Morus*) *Morus wittiorum*	Tropical and Subtropical regions of Asia, Africa, South America	2014	Anti-oxidant activity	Potentially effective antioxidants	59
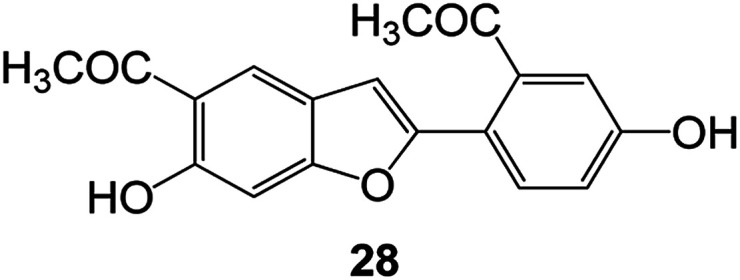	(Leguminosae) *Mucuna birdwoodiana*	Southern China	2009	Anti-oxidation	Potentially effective antioxidants	60
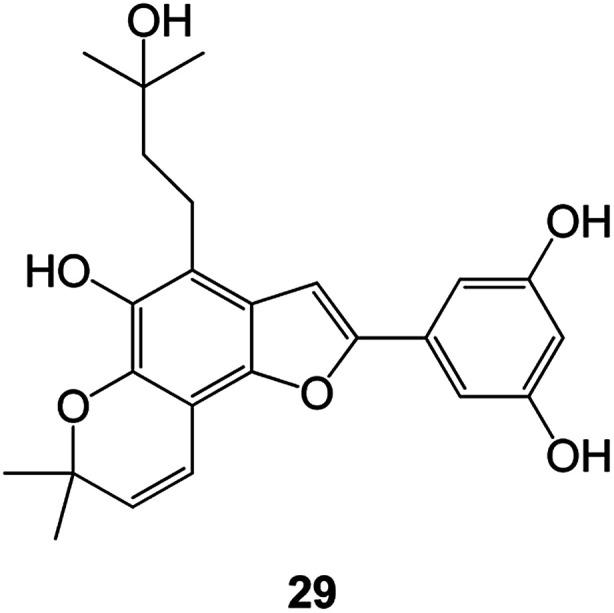	(*Morus*) *Morus wittiorum*	Southern China	2010	Anti-inflammatory and anti-oxidation	As a multi-target potential anti-inflammatory drug	61
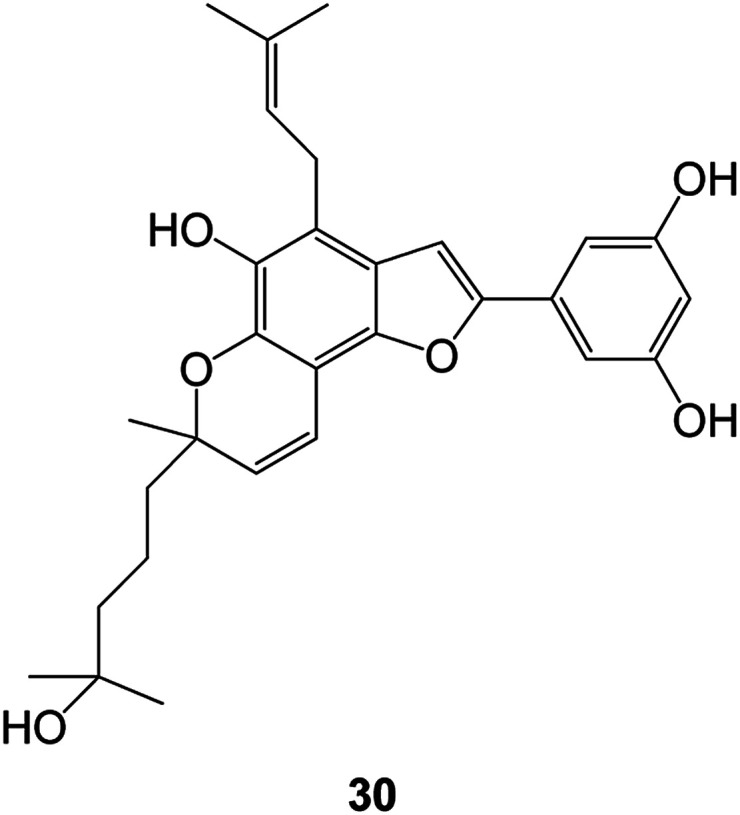						
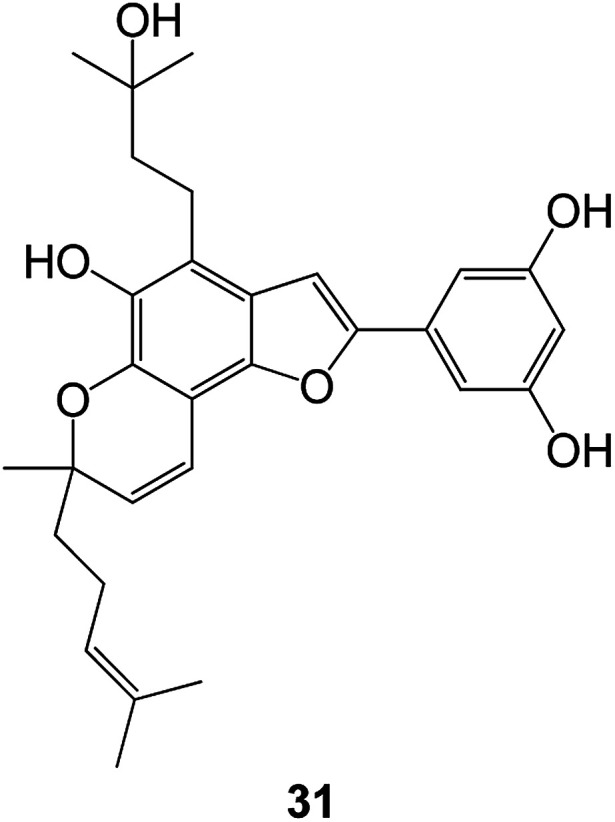	(*Morus*) *Morus wittiorum*	Southern China	2010	Anti-inflammatory and anti-oxidation	As a multi-target potential anti-inflammatory drug	61

## 4. Activity of benzofuran derivatives

Many benzofuran compounds, including those isolated from plants as well as obtained by synthesis, exhibit a variety of biological activity.^62^ In recent years, these compounds have received widespread attention from researchers. The literature shows that compounds with benzofuran nucleus have a wide range of therapeutic potentials, including antibacterial, antifungal, anti-inflammatory, analgesic, anti-depressant, anticonvulsant, anti-tumor, anti-HIV, anti-diabetic, anti-tuberculosis, anti-oxidation, among others activities.^63^ Many efforts have been made to achieve efficient synthesis of these motifs.^64^ The synthesis of benzofuran can be divided into phenol, benzoic acid and miscellaneous methods according to the raw materials of the reaction.^65^ Preliminary studies on the structure–activity relationship of benzofuran compounds showed that the ester group at the C-2 position is a key site for the cytotoxic activity of the compounds, and the introduction of heterocyclic substitution at the C-2 position also has a certain effect on cytotoxicity. The introduction of the substituents at the 2-position phenyl group and the 5-position hydroxyl group, halogen, and amino group is closely related to the antibacterial activity of the benzofuran. Here we review the structure and biological activities of benzofuran compounds and their synthesis methods. The benzofuran derivates in this review are classified according to their biological activities.

### 4.1 Antitumor activity

Malignant tumors are one of the major diseases that threaten human health. Nowadays, the mortality rate of malignant tumors is second only to cardiovascular disease.^66^ Therefore, finding novel and effective anticancer drugs and treatment methods to overcome malignant tumors is an important research topic in the field of medicine and pharmacy.^67^ With the continuous development of related research, the anti-tumor activity of benzofuran compounds has attracted more and more attention from scientists in recent years.

**Fig. 6:**
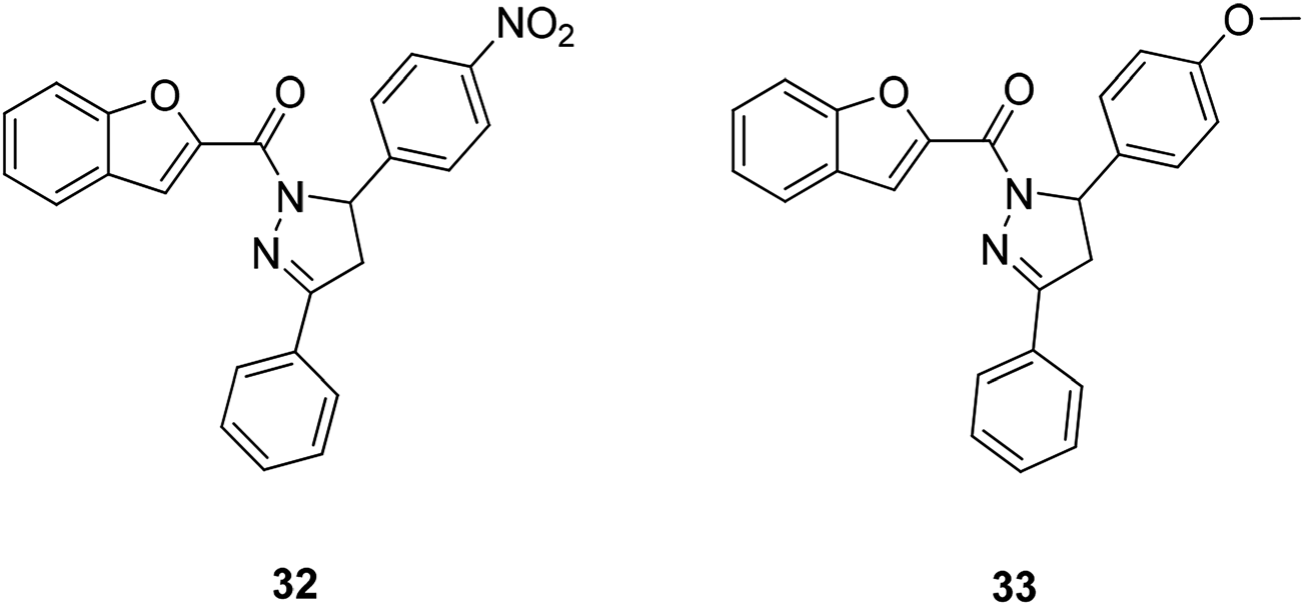
Benzofuran derivatives 32 and 33 active against ovarian cancer.

A series of benzofuran-2-yl-(4,5-dihydro-3,5-substituted diphenylpyrazol-1-yl)methanone compounds have been obtained by microwave-assisted synthesis (MWI). The anticancer activity of this class of compounds against the human ovarian cancer cell line A2780 was evaluated. Compound 32 (IC_50_ = 12 μM) and compound 33 (IC_50_ = 11 μM) (Fig. 6) were found to be the most active ones among this series of compounds.^68^

**Fig. 7:**
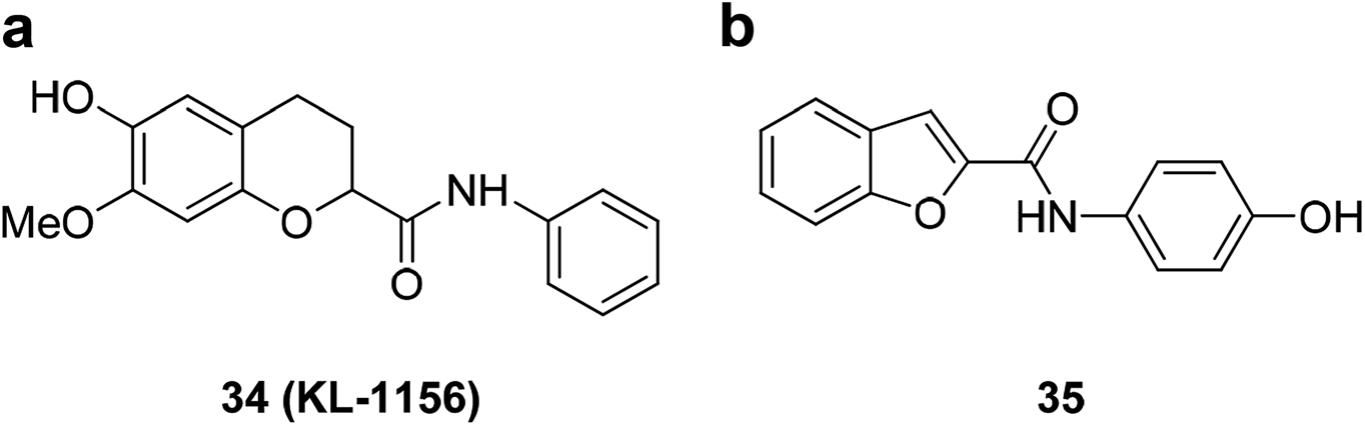
(a) Structure of the lead compound 34 (KL-1156). (b) Compound 35 exhibiting excellent anticancer and NF-κB inhibitory activity.

A series of new benzofuran- and 2,3-dihydrobenzofuran-2-carboxylic acid *N*-(substituted) phenylamide derivatives were designed and synthesized from the lead compound 34 (KL-1156). Among them, compound 35 (Fig. 7) showed significant growth inhibitory activity against a series of cancer cell lines with GI_50_ values of 2.74 μM (ACHN), 2.37 μM (HCT15), 2.20 μM (MM231), 2.48 μM (NUGC-3), 5.86 μM (NCI-H23) and 2.68 μM (PC-3).^69^ Compound 35 also showed excellent NF-κB inhibitory activity.^70^

**Fig. 8:**
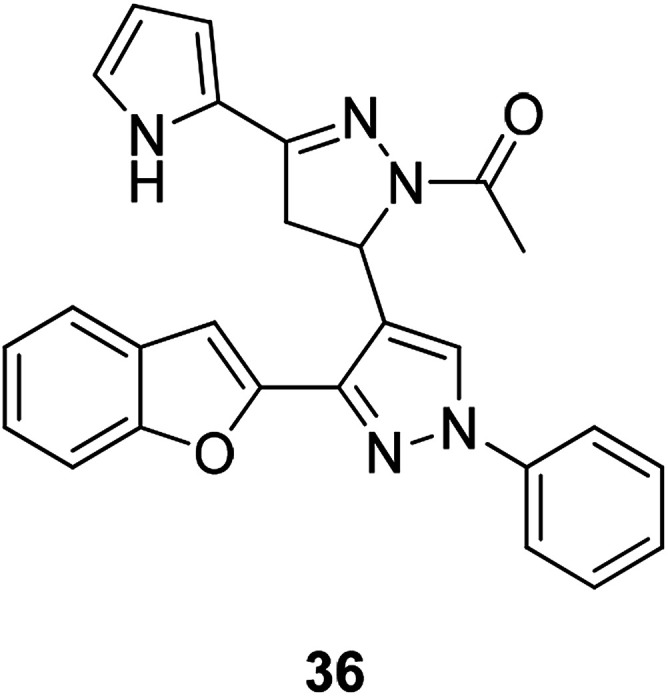
A benzofuran-pyrazole derivative compound 36 having good anticancer activity.

Development of promising compounds with target therapy potentials and little side effects is the main goal of medical researchers.^71^ Literature has shown that some substituted benzofurans have dramatic anticancer activities.^71^ Compound 36 (Fig. 8) was found to have significant cell growth inhibitory effects, and the inhibition rates in different types of cancer cells by 10 μM of compound 36 are as follows: Leukemia K-562 and SR (inhibition rate: 56.84% and 60.89% respectively), Non-small cell lung cancer NCI-H322M and NCI-H460 (inhibition rate: 40.87% and 80.92% respectively), Colon cancer HCT-116, KM12 and SW-620 (inhibition rate: 72.14%, 41.49 and 40.82% respectively), CNS cancer SNB-75 and U251 (inhibition rate: 58.02% and 73.94% respectively), Melanoma LOX IMVI and MDA-MB-435 (inhibition rate: 72.69% and 50.64% respectively), and Ovarian cancer OVCAR-4 and OVCAR-8 (inhibition rate: 56.45% and 44.50% respectively). Compound 36 (10 μM) also produced a significant inhibitory on Src kinase (inhibition rate: 59%). These results indicate that compound 36 has good anticancer activity.

**Fig. 9:**
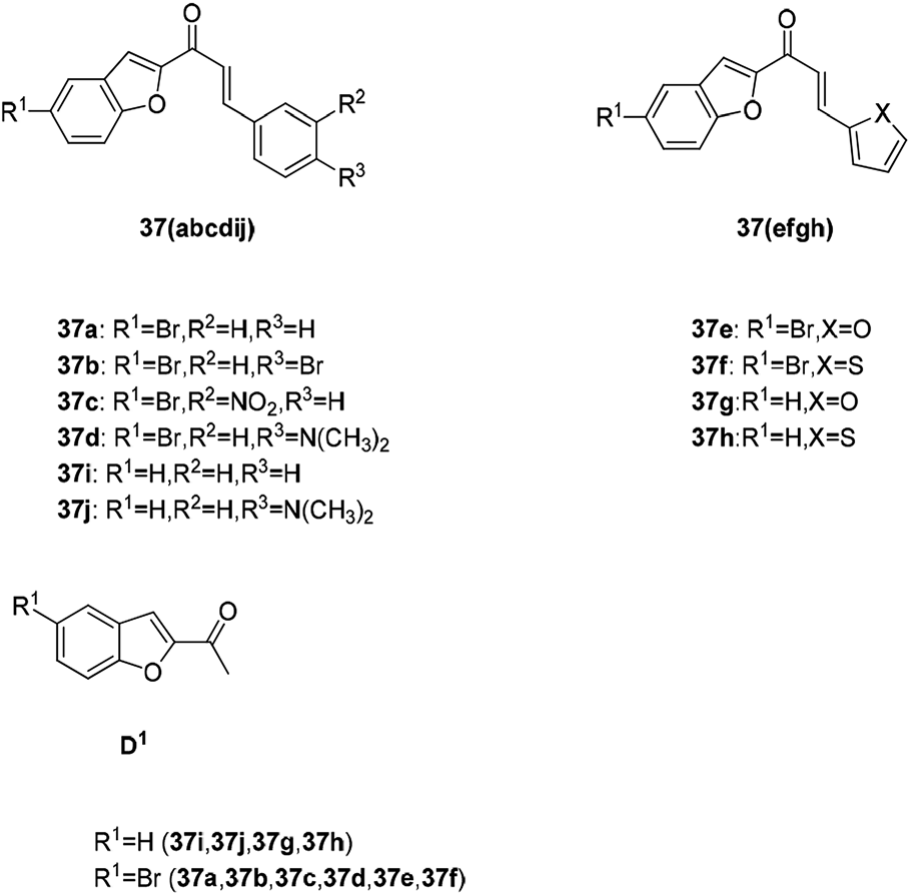
The structure of the chalcone derivatives 37a–37j and benzofuranone D^1^.

Benzofuran substituted chalcone compounds are also important anticancer drug research directions in recent years. A novel series of chalcones, 3-aryl-1-(5-bromo-1-benzofuran-2-yl)-2-propanones propenones (37a–f), were designed, synthesized, and characterized (Fig. 9). The *in vitro* antitumor activities of the newly synthesized (37a–f) and previously synthesized (37g–j) chalcone compounds were determined by using human breast (MCF-7) and prostate (PC-3) cancer cell lines.^72^ The structure and activity comparison between these chalcone derivatives and the starting material (D^1^) in Table 2 indicates that the benzofuran-substituted chalcone exhibits a better activity than the raw material with only the unsubstituted benzofuran ring (D^1^).

**Table 2:** Evaluation of the cytotoxicity and log IC_50_ values (μM) of chalcone compounds and docetaxel (reference chemotherapeutic drug) on two cancer cell lines^a^

Compound	MCF-7 log IC_50_ (μM)	PC-3 log IC_50_ (μM)
D^1^	2.12	1.54
37a	1.89	1.67
37b	1.15	1.24
37c	5.01	6.31
37d	5.79	1.81
37e	0.42	0.67
37d	2.30	2.47
37f	4.43	6.11
37g	2.55	2.57
37h	6.28	6.30
37i	−0.21	0.92
Docetaxel (reference drug)	−0.52	−0.52

a All the compounds at 100 μM significantly reduced the viability of PC-3 and MCF-7 cells (*p* < 0.001).^66^

**Fig. 10:**
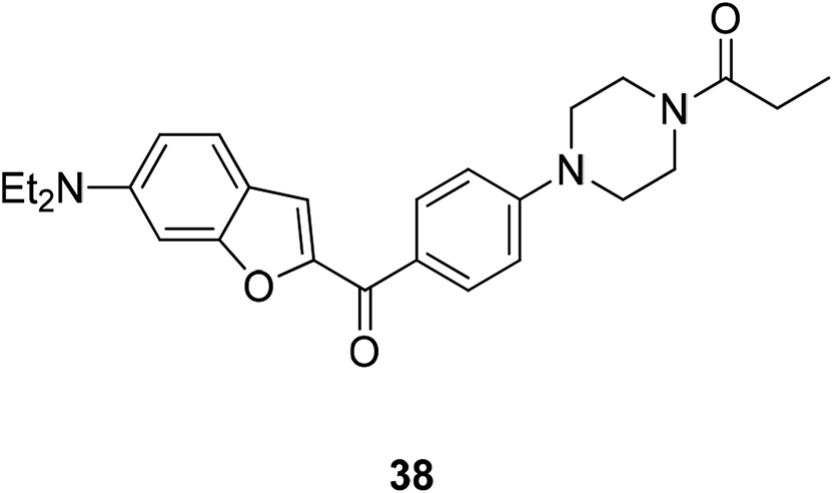
Structure of compound 38 having anti-inflammatory, anti-tumor, and antioxidant biological activities.

Recently some new benzofurans with *N*-aryl piperazine derivatives were discovered or synthesized. These compounds have been identified in a series of *in vitro* screening models to exhibit good activities especially anti-inflammatory and anti-cancer activities. The results showed that compound 38 (Fig. 10) inhibited NO production (IC_50_ = 5.28 μM) and selectively inhibited the proliferation of human lung cancer cell line (A549) and gastric cancer cells (SGC7901) (IC_50_ = 0.12 μM and 2.75 μM, respectively); therefore compound 38 is an excellent anti-inflammatory and anti-tumor drug.^64^

**Fig. 11:**
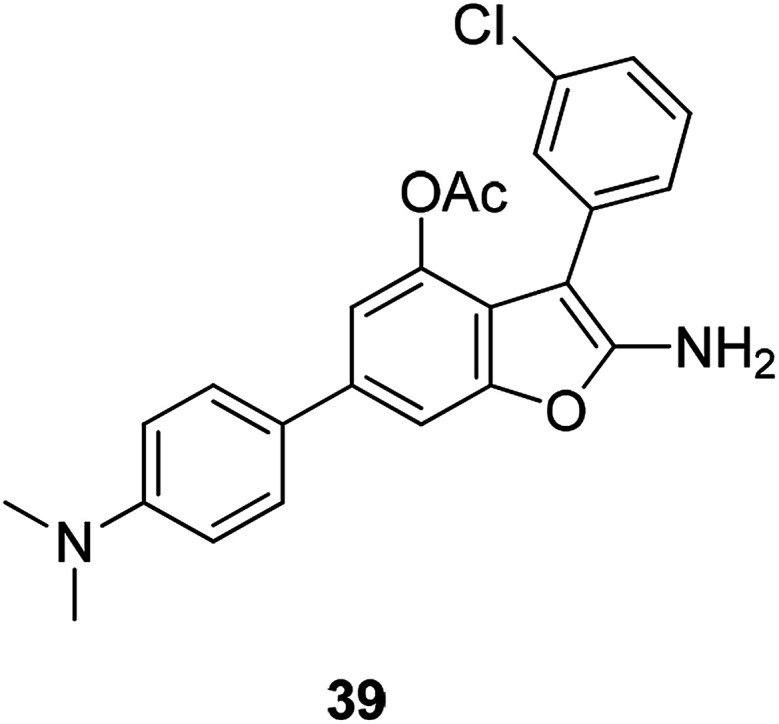
The structure of the compound 2-amino-3-(2-chlorophenyl)-6-(4-dimethylaminophenyl)benzofuran-4-yl acetate (39).

Chondrosarcoma is a bone tumor with high mortality and has weak responsiveness radiation therapy and chemotherapy. The newly synthesized attractive lead compound 39 (Fig. 11) showed good pharmacological properties in terms of anti-human chondrosarcoma. It can be further developed as a lead compound for anti-human chondrosarcoma.^73^

### 4.2 Antimicrobial activity

#### 4.2.1 Anti-bacterial

The research of antifungal drugs has a history of nearly 100 years. Traditional benzofuran drugs such as imidazole,^74^ pyrimidine,^75^ and triazole^10,76,77^ and antibiotics and propionamides, have been widely applied clinically; and they all show good antibacterial activities. However, in recent years, the drug abuse, the emergence of new immunosuppressive agents, as well as clinical radiotherapy, chemotherapy, and organ transplantation all led to impaired immune system function and dysbacteriosis, resulting in decreased immunity of the body, eventually leading to an increase in fungal infection rate. In addition, since both fungi and human cells are eukaryotic cells, they are usually toxic to host cells after long-term administration. Therefore, the immunity of pathogenic fungi to existing antifungal drugs becomes a more serious problem. Seeking effective and low-toxic antifungal drugs has become the focus of current research. Pharmaceutical chemists have found that benzofuran and its derivatives are very suitable for this because they exist widely in natural products and have a wide range of biological and pharmacological activities; therefore, benzofurans have drawn considerable attention in this field.^78^

**Fig. 12:**
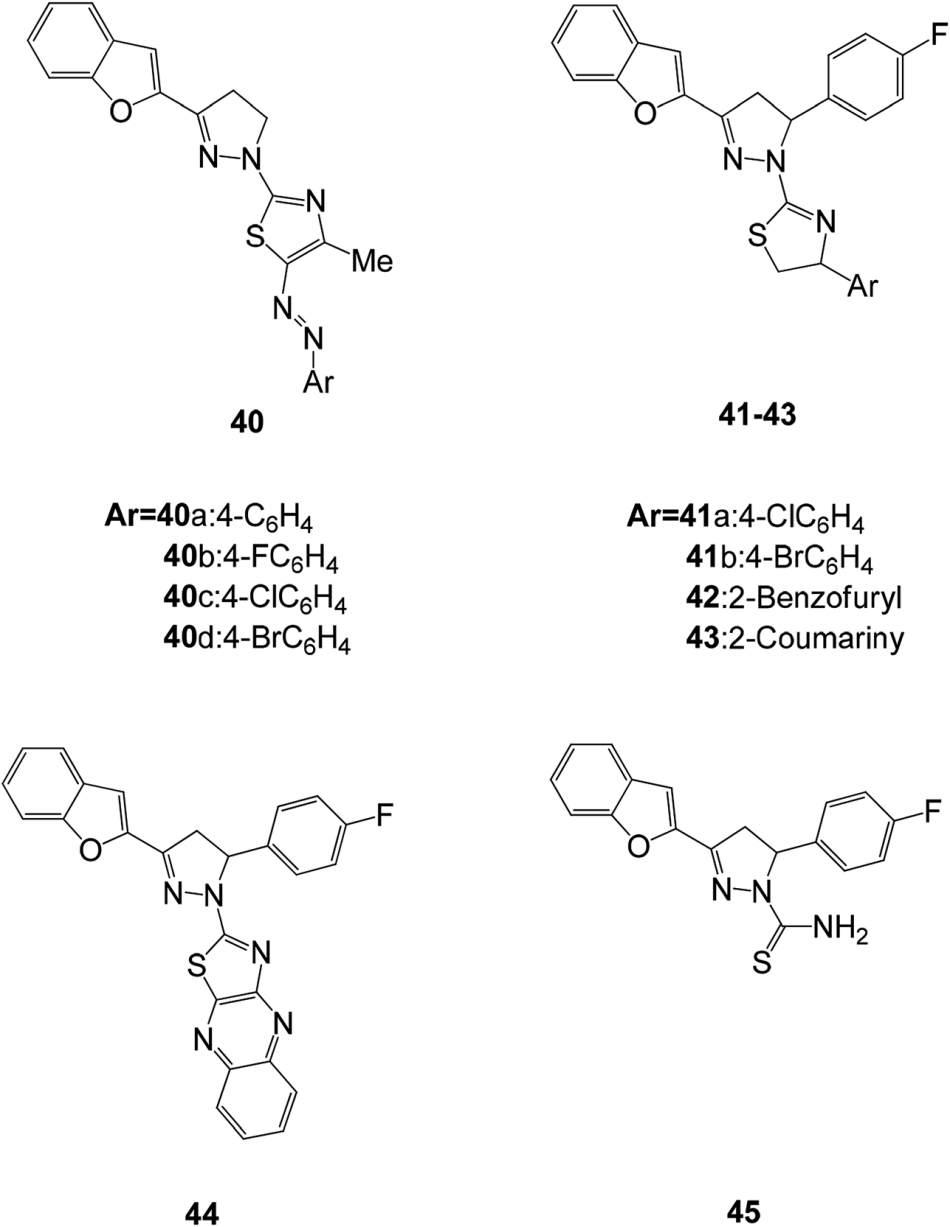
Structures of fluorinated 2-(3-(benzofuran-2-yl)pyrazol-1-yl)thiazoles.

Mohamed *et al.* synthesized a series of benzofuran-based pyrazoline-thiazoles 40(a–d) and fluorinated pyrazole-thiazole (41–43) derivatives and tested their potential antimicrobial activities against four Gram-positive bacteria (*Staphylococcus aureus* (*S. aureus* (*SA*)), *Bacillus subtilis* (*B. subtilis* (*BS*)), *Bacillus megaterium* (*B. megaterium* (*BM*)), and *Sarcina lutea*) and Gram-negative bacteria (*Klebsiella pneumoniae* (*K. pneumoniae* (*KP*)), *Pseudomonas aeruginosa* (*P. aeruginosa* (*PA*)), and *Escherichia coli* (*E. coli* (*EC*))).^79^ Among all the compounds tested, 40c showed excellent antimicrobial activity compared to the control drug (ciprofloxacin and ketoconazole) and had inhibitory activity against most microorganisms.^80^ Compounds 40b and 40d suppressed the growth of S. aureus with inhibition zones (IZ) of 23 and 20 mm, respectively, while compound 41a showed promising antifungal activity against *K. pneumoniae*, *P. aeruginosa*, and *E. coli* with an IZ of about 24 mm. Structure activity relationship studies have shown that the antibacterial activity of these compounds is closely related to the presence of chloro substituents on the pyrazoline and pyrazole moieties (40c and 41a) (Fig. 12).

**Fig. 13:**
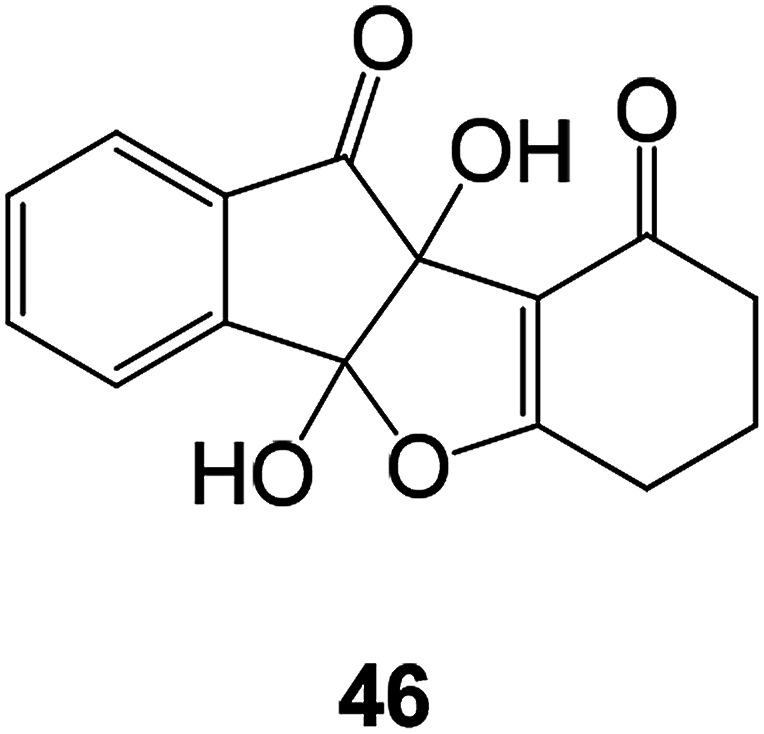
Structure of compound 46.

In order to develop new antibacterial benzofuran compounds Sayed Hasan Mehdi synthesized compound 46 and evaluated its biological activity using microdilution method against Gram-positive (*B. subtilis*, *B. cereus*, *S. pneumoniae*, and *S. aureus*) and Gram-negative (*K. pneumoniae*, *S. flexneri*, *P. aeruginosa*, *E. aerogenes*, and *E. coli*) bacterial and fungal (*C. albicans*) strains^81^ The MIC value of compound 46 tested microorganisms were found to be between 0.5 and 1 mg mL^−1^, comparable to the MIC value of the clinically used antimicrobial agents (Fig. 13).^82^

**Fig. 14:**
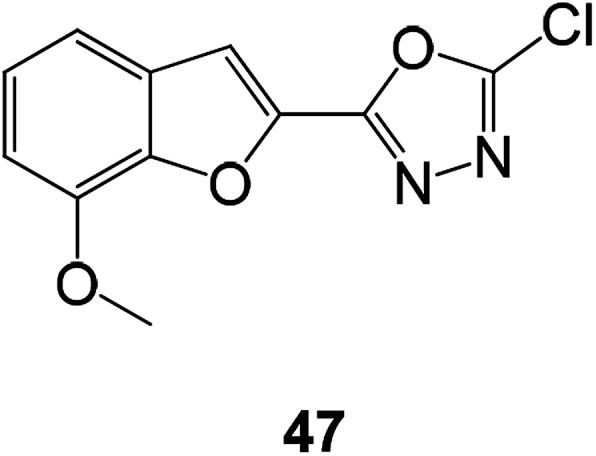
Structure of compound 47.

A series of compounds containing benzofuran ring with oxadiazoles and pyrazoles were constructed and tested against five bacterial (*E. coli*, *K. pneumonia*, *P. aeruginosa*, *S. aureus*, and *Streptococcus faecalis*) and five fungal strains (*A. flavous*, *A. fumigatus*, *C. albicans*, *Penicillium notatum*, and *Rhizopus*). Among them, compound 47 showed strong activities against all the tested microbial species (Fig. 14).^62^

**Fig. 15:**
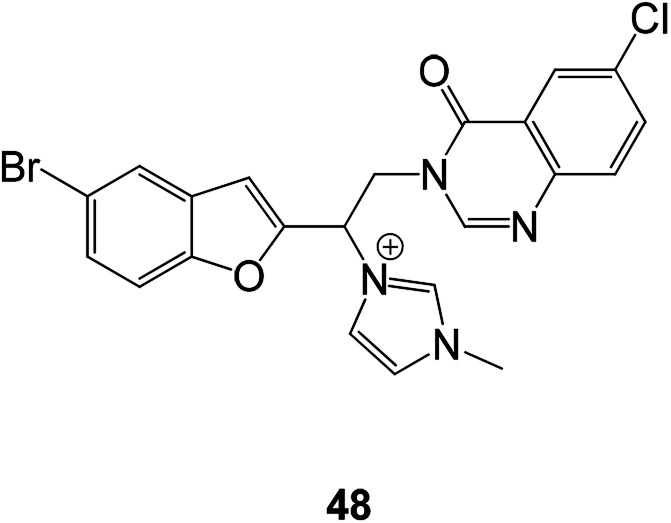
Structure of novel antibacterial compound 48.

Some benzofuran derivatives linked to other heterocycles (quinazolines) have been used in antibacterial research in recent years. The antibacterial activities of these synthesized compounds were evaluated against three Gram-negative (*Escherichia coli*, *Pseudomonas aeruginosa* and *Saμmonella typhi*), three Gram-positive (*Staphylococcus aureus*, *Bacillus subtilis* and *Listeria monocytogenes*) and one yeast-like fungi (*Candida albicans*). Among them, compound 48 showed the highest antimicrobial activity against all the tested strains (Fig. 15).^83^

**Fig. 16:**
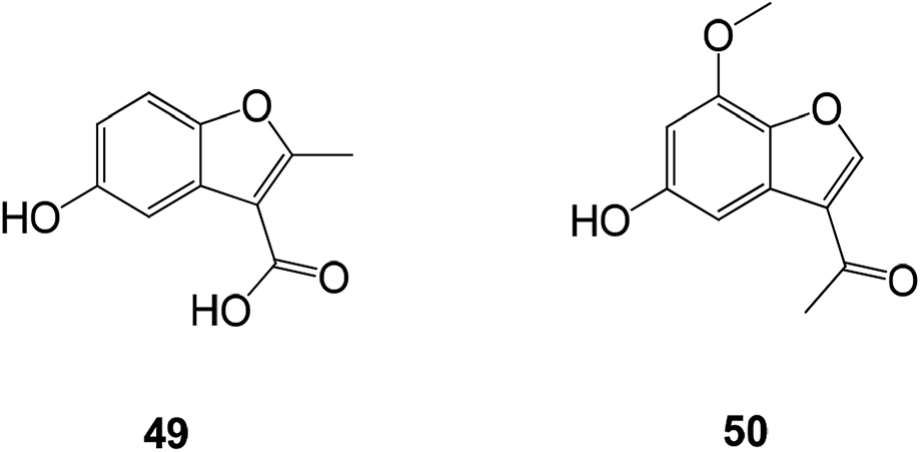
Structures of the isolated compounds 49 and 50.

Talaromyces amestolkiae YX1 is a marine fungal strain obtained from the fresh tissue of the marine mangrove plant *Kandelia obovata*. The compound obtained by extracting the fungal fermentation broth using EtOAc was shown to have an inhibitory effect on the activity of α-glucosidase. Two novel benzofuran compounds were obtained by further fractionating the biologically active extract (Fig. 16).^84^

**Fig. 17:**
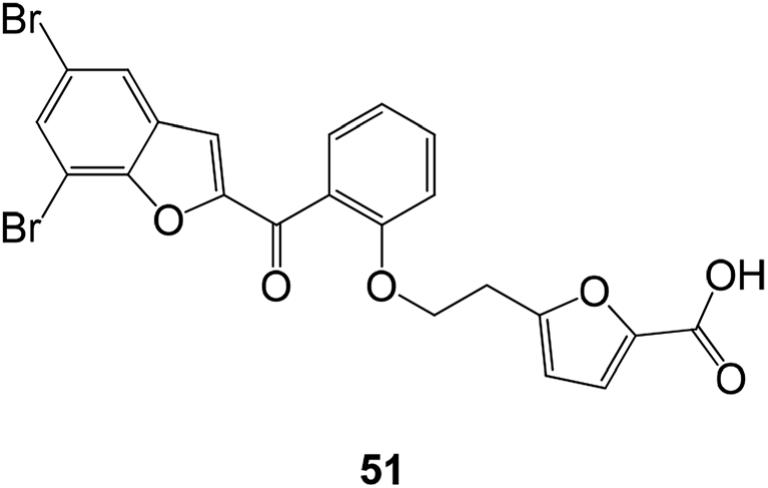
Design of novel 2-salicyloylbenzofuran derivatives as antibacterial agents.

The 2-salicylidene benzofuran derivatives showed an antibacterial effect. Compound 51 showed the most potent antibacterial activity with MIC values of 0.06–0.12 mM against three Gram-(+) bacterial strains (Fig. 17).^85^

**Fig. 18:**
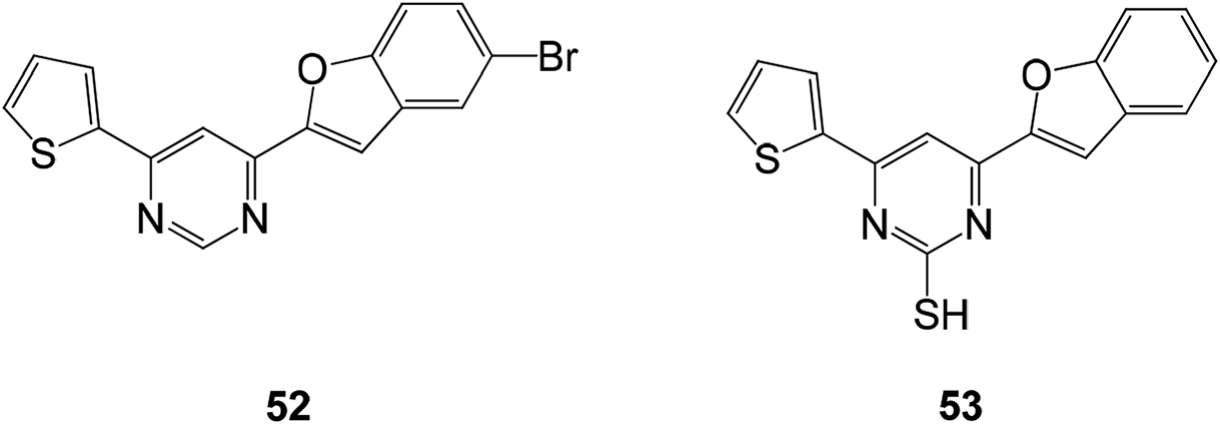
Structure of benzofuran pyrimidine derivatives 52 and 53.

Biologically active benzofuran compounds containing pyrimidine ring were constructed from benzofuran chalcones which have high chemical reactivity and diverse synthetic applications. Antimicrobial activity test results showed that the presence of hydroxyl, thiol, and amino groups in the pyrimidine ring significantly contributes to their antimicrobial activities (Fig. 18).^86^

#### 4.2.2 Antiviral activity

**Fig. 19:**
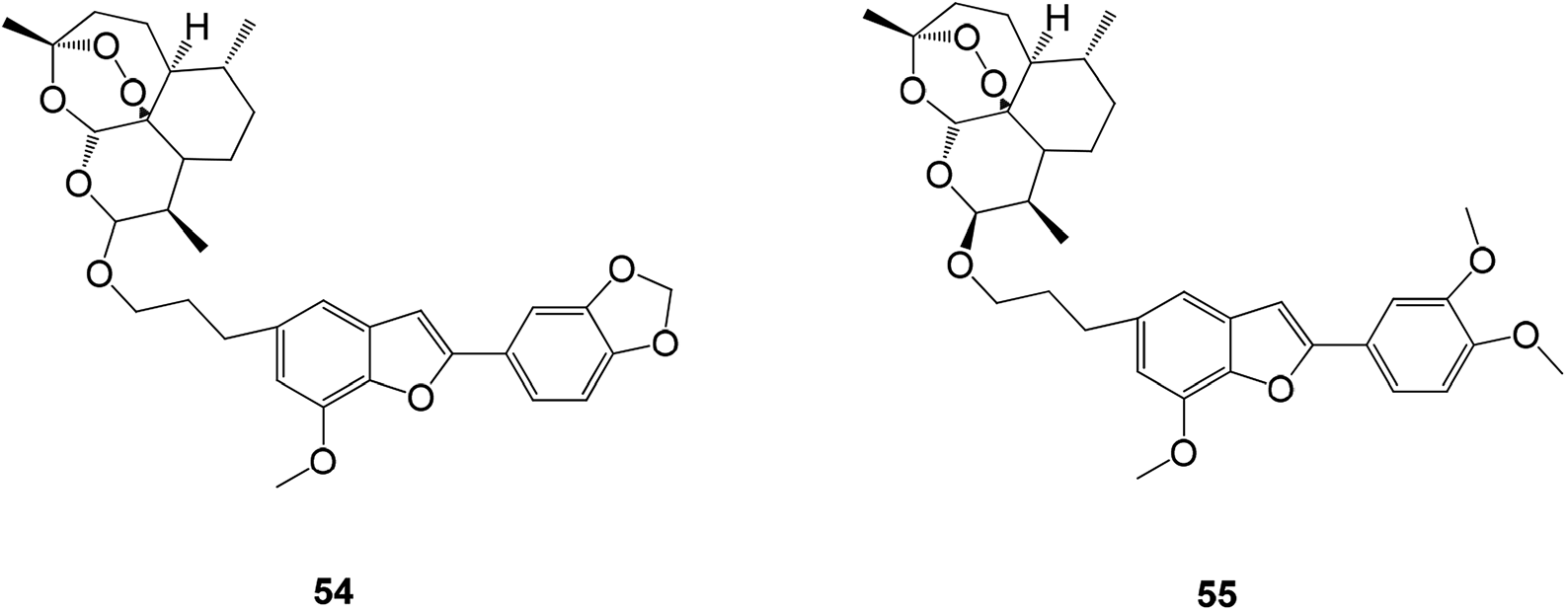
The structure of compounds 54 and 55 with inhibition of HCMV Mars.

Viruses are an important class of pathogens harmful to human health. They are highly contagious and can lead to far-reaching harm to many other diseases (such as cancer). Viral structure is very simple, and intracellular parasites and viral replication depend on the characteristics of the host cell, which results in most antiviral drugs being more toxic to the body or having a lower antiviral effect when used therapeutically. This is one of the reasons for the slow development of antiviral drugs. New benzofuran antiviral drugs were obtained by hybridization of natural products (homologous) egonol, thymol and artemisinin. Some hybrid compounds are also potential antiviral drugs due to their excellent anti-HCMV activity in the micromolar to sub-micromolar range, wherein the ether-linked artemisinin-homologous triol mixture 55 exhibits the strongest anti-HCMV activity with an EC_50_ value of 0.13 μM. It is 20 times more active than the control drug ganciclovir. Hybrid 54 was linked by another ether consisting of egonol and dihydroartemisinin with an EC_50_ value of 0.17 μM (Fig. 19).^87^

**Fig. 20:**
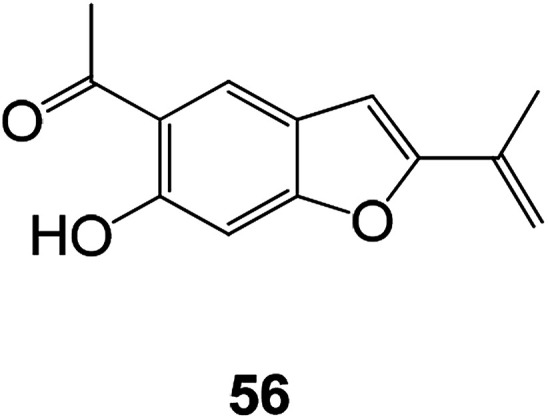
Chemical structures of 56.

Compound 56 is a benzofuran derivative having antiviral activity found in *Eupatorium adenophorum* which exhibits antiviral effects against RSV LONG and A2 strains with IC_50_ values of 2.3 and 2.8 μM, respectively. The results of 3-(4,5-dimethylthiolan-2-yl)-2,5 diphenyltetrazolium bromide (MTT) assay showed that compound 56 is cytotoxic and has a 50% cytotoxicity (CC_50_) value of 7.9 μM. Although it is considered to have a lower selection index (SI, ratio of CC_50_ to IC_50_) of 3.4 and 2.8, respectively, it can be used as an antiviral drug to optimize drug selectivity (Fig. 20).^88^

**Fig. 21:**
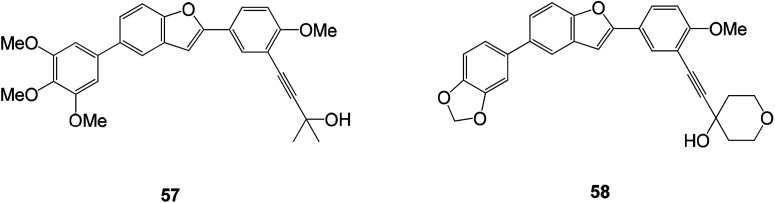
Structure of compounds 57 and 58 of benzofuran analogs of HCV inhibitors.

Benzofuran-type hepatitis C virus inhibitors were discovered recently through high-throughput screening. Shanshan He and co-workers discovered compounds 57 and 58, which showed promising antiviral activities. Notably, it was observed that such compounds were able to significantly reduce intracellular viral levels, suggesting that compounds 57 and 58 may be effective against early infection of HCV. At the same time, the cytotoxicity of the compounds was further determined in HepG2 cells and primary human hepatocytes, and the compounds showed low cytotoxicity for both types of cells. The experimental results also indicate that one of the advantages of this series of antiviral compounds is their low cytotoxicity to human hepatocytes (CC_50_ > 31.6 μM) (Fig. 21).^89^

### 4.3 Antioxidant activity

**Fig. 22:**
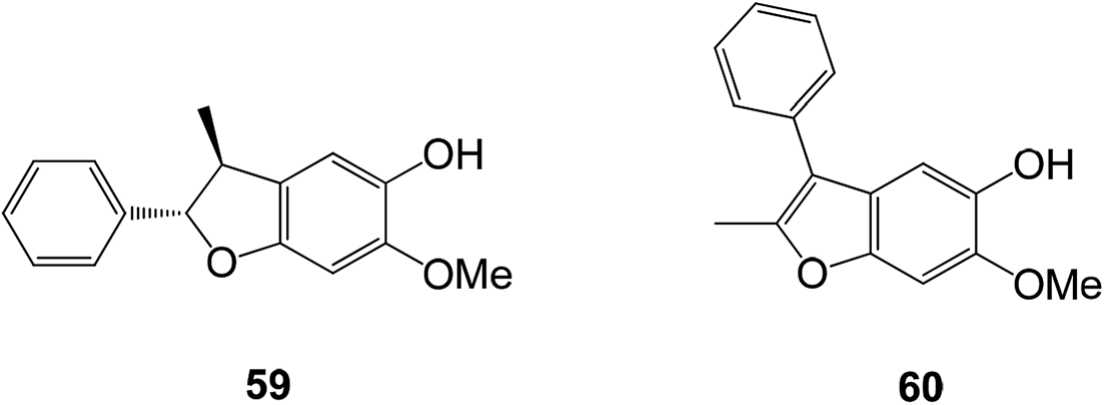
Chemical structures of compounds 59 and 60.

Free radicals produced by normal biochemical reactions in the body play an important role in the human body and become harmful only when they are produced in large quantities. The human body has an innate defense mechanism to resist free radicals such as superoxide dismutase, catalase and glutathione peroxidase.^90^ Studies have found that benzofuran compounds have good antioxidant activity (Fig. 22).^91,92^

A new line of benzofuran compounds 59 and 60 were isolated from *D. latifolia*. These compounds had moderate antioxidant activity and were tested by DPPH free radical scavenging test with an IC_50_ value of 96.7 ± 8.9 μM.^93^

**Fig. 23:**
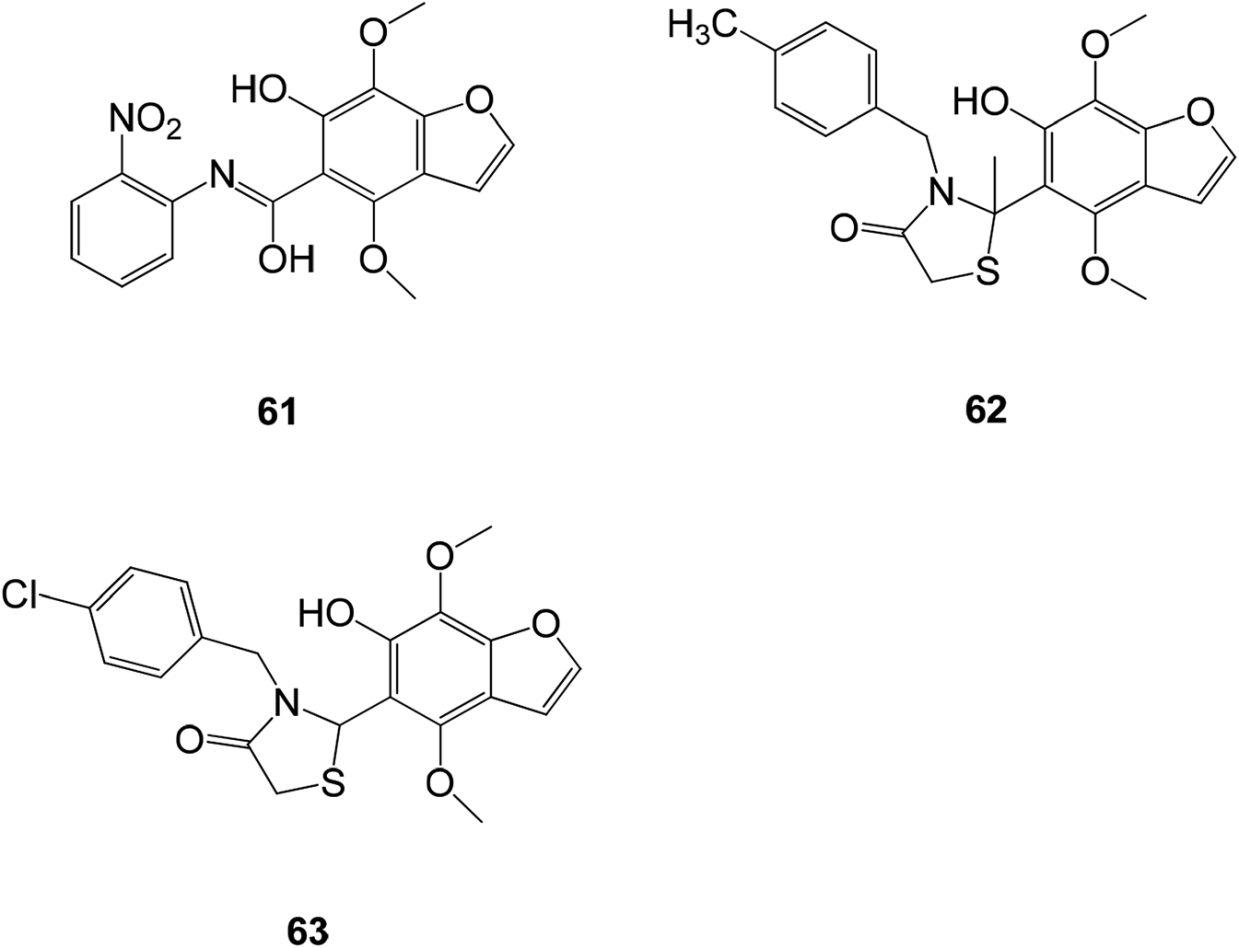
Structures of the compounds (61–63).

The flavonoids and benzofurans have been identified as novel antioxidants in medicinal chemistry and have become a new research direction for antioxidants. The 1,3-benzofuran derivatives (Fig. 23) (61–63) have very similar antioxidant activities with EC_50_ values of 8.57, 9.72, 8.27 and 10.59 mM, respectively.^1^

**Fig. 24:**
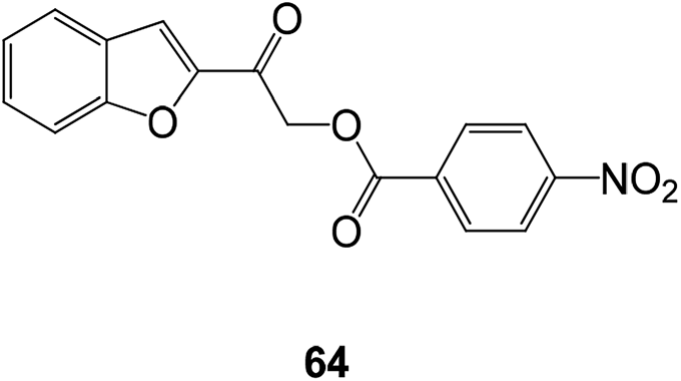
Chemical structure of benzofuran ester compound 64 having antioxidant activity.

Benzofuran esters are also an important development direction of antioxidant drugs. A series of new benzofuran ester compounds have been synthesized and their activity has been detected. Results showed that a compound 64 (DPPH) has the highest free radical scavenging activity (Fig. 24).^94^

**Fig. 25:**
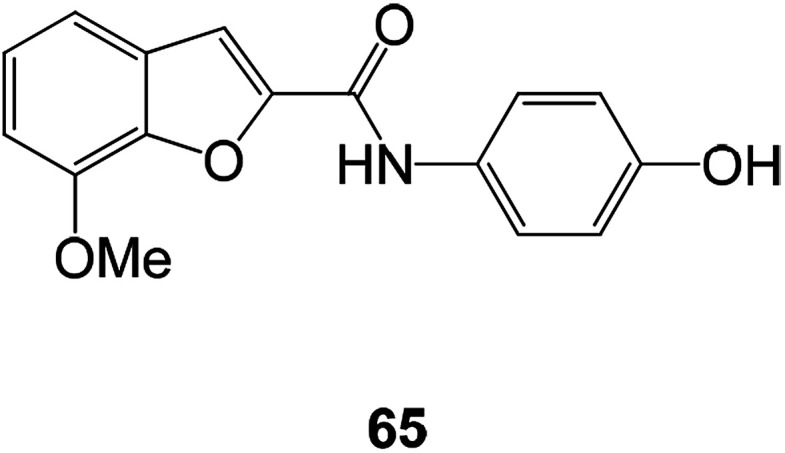
The structure of the 7-methoxy-*N*-(substituted phenyl)benzofuran-2-carboxamide derivative.

The novel benzofuran-2-carboxamide derivatives also exhibited certain antioxidant activity. Compound 65 exhibited moderate to appreciable antioxidant activity. At 100 μM, the inhibition rate on LPO was 62%, and the inhibition rate on DPPH radical formation was 23.5% (Fig. 25).^95^

The substituted benzofuran derivative 66 was tested with the antioxidant l-ascorbic acid as a positive control, and the antioxidant activity tests at concentrations of 200, 100 and 50 μg mL^−1^ showed that the compounds 66a–66g had excellent antioxidant activity (Table 3).^96^

**Table 3:** Structure and antioxidant activity data of compounds 66(a–g)

Compounds	R^1^	R	Concentration in (μg mL^−1^)		
			200	100	50
66a (ref. 96)	Morpholine	Br	95.3	95.3	91.9
66b (ref. 96)	Morpholine	OMe	100	95.3	88.1
66c (ref. 96)	*N*-methyl piperazine	OMe	94.6	80.2	28.7
66d (ref. 96)	Thiomorpholine	Br	98.1	91	89.8
66e (ref. 96)	1-Pyridyl-2-ylpiperazine	Br	97.9	94	85.7
66f (ref. 96)	1-Pyridyl-2-ylpiperazine	OMe	100	97.2	93.6
66g (ref. 96)	2-Piperzin-1-ylethanol	OMe	94.2	93.2	92.6
l-Ascorbic acid			99.2	99	98.8

### 4.4 Anti-AD activity and synthesis of benzofuran derivatives

AD is one of the most common neurodegenerative diseases and is ranked as the fourth most common disease among the elderly and an important cause of death in the elderly. Alzheimer's disease is a type of neurological disease that has attracted more and more attention in recent years.^97^ Many pharmaceutical companies have used classical methods to find new drugs for treating AD through different stages of clinical trials. In recent years, more than a dozen of different drugs have been tested clinically and have not shown positive results or efficacy.^98^ Benzofurans and terpenoids as inhibitors of butyryl cholinesterase (BuChE), acetylcholinesterase (AChE), gamma secretase, β-secretase, tau misfolding and β-amyloid (Aβ) aggregation, have good effects in the treatment of AD.^99^

**Scheme 1:**
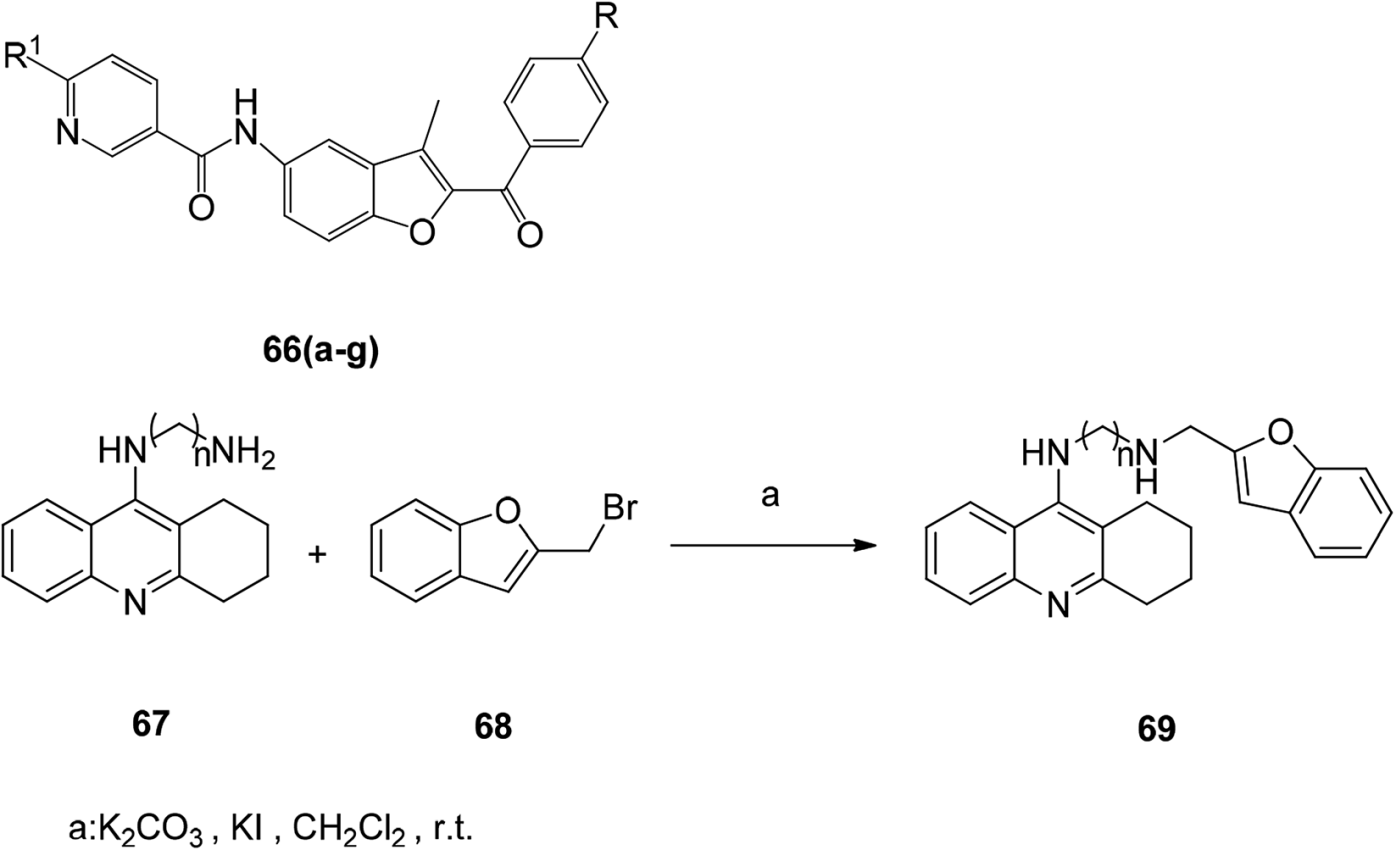
Synthesis of compound 69.

The novel tacrine-benzofuran hybrids were synthesized and evaluated on key molecular targets of AD (Scheme 1). Most hybrids exhibit good inhibitory activity against cholinesterase and Aβ self-aggregation. Selected compounds showed significant inhibition of human β-secretase-1 (hBACE-1). Among these hybrids, compound 69 showed the best activity as a good inhibitor of subnanomolar selective human acetylcholinesterase (hAChE) (IC_50_ = 0.86 nM) and Aβ aggregation (hAChE- and self-induction, 61.3% and 58.4% respectively) and hBACE-1 activity (IC_50_ = 1.35 μM).^100^

### 4.5 Anti-inflammatory activity of benzofuran derivative and its synthesis

**Scheme 2:**
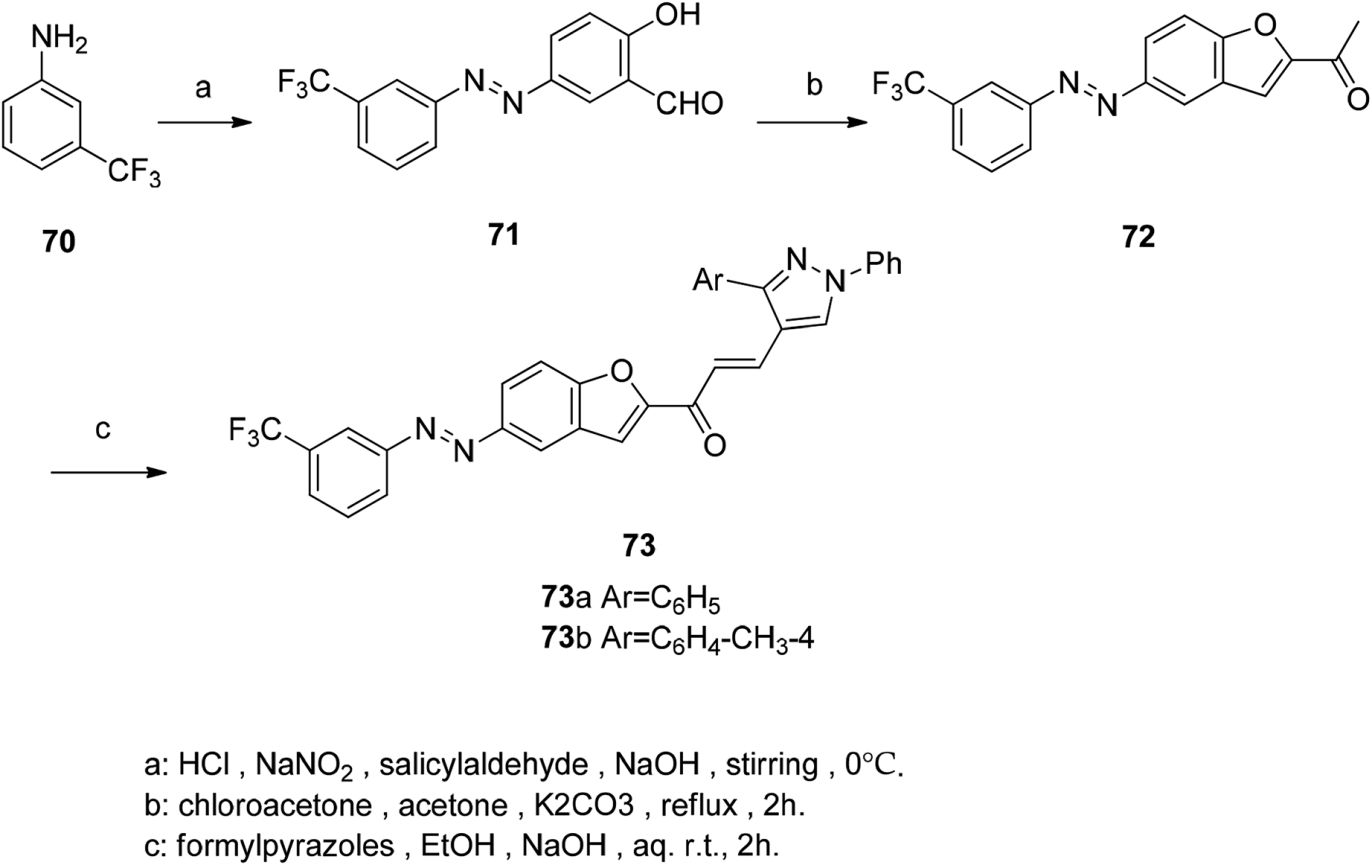
Synthetic route for acetyl benzofuran 73.

In multicellular organisms, inflammation is the primary host defense response to tissue damage, infectious agents or autoimmune responses, and is also an important component of the immune response. Symptoms of inflammation include swelling, redness of the skin, and pain. It can be acute or chronic depending on time and pathological characteristics.^101^ In recent years, studies on benzofuran derivatives have found that individual derivatives have good anti-inflammatory activity. The core compound 2-acetylbenzofuran was synthesized by coupling 3-(trifluoromethyl) phenyl diazo chloride with salicylaldehyde. Then cyclization condensation reaction was carried out with 5-((3-(trifluoromethyl)phenyl)diazenyl)salicylaldehyde and alkenyl salicylaldehyde.^102^ The acetyl group in compound 72 is a general precursor for the synthesis of chalcone and pyrazoline derivatives, and therefore anhydrous potassium carbonate must be present in the reaction. Claisen–Schmidt condensation of 2-acetylbenzofuran with formylpyrazole gives the corresponding chalcone derivatives 73a and 73b, which are compounds with good anti-inflammatory activity (Scheme 2).

### 4.6 Vasorelaxant agents

**Fig. 26:**
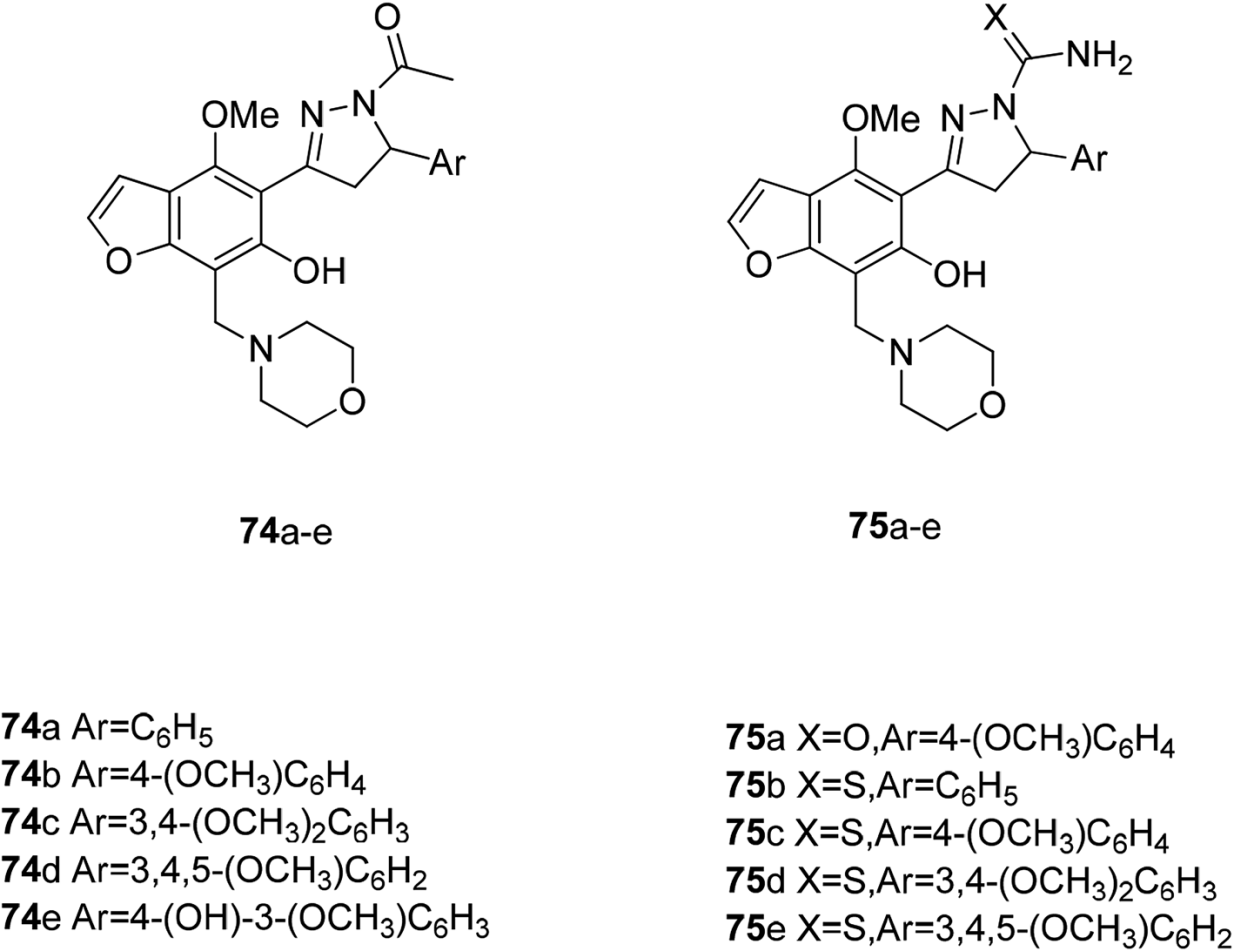
Some benzofuran–morpholinomethyl–pyrazoline hybrids with vasodilating activity.

Vasodilators are a class of smooth muscle relaxants that have the effect of dilating blood vessels. They are mainly used to treat high blood pressure, heart failure and angina. Benzofurans are promising candidates because of their antiarrhythmic, hypotensive, and vasodilating effects. At the same time, the morpholine scaffold is a very versatile structure in the drug design process and has a variety of biological and pharmacological activities. A novel vasodilating compound may be provided by the combination of these two active structures. Among the several types of compounds designed by the principle of active molecular assembly, the *N*-acetylpyrazoline derivative exhibits the highest activity, and the pyrazoline thiocarboxamide also has a good activity. The results for *N*-acetylpyrazoline 75a–e showed that compounds 75a–c (IC_50_ 0.4171, 0.4550 and 0.3704 mM) showed better activity than prazocin, while the activity of compound 74d (IC_50_ 0.4951 mM) was comparable to that of prazocin and compound 74e (IC_50_ 0.5340 mM), and was slight less than that of prazocin. In the case of pyrazoline, the carboxamide derivatives 75a and c (IC_50_ 0.4475 and 0.4158 mM, respectively) showed better activity than prazocin. The thiosulfamide derivatives 75b, d and e (IC_50_ 0.4212, 0.4041 and 0.3505 mM) showed better activity, and the compound 75c (IC_50_ 0.5815 mM) showed a lower activity than the prazocin (Fig. 26).^103^

## 5. General synthesis method of benzofuran

**Scheme 3:**
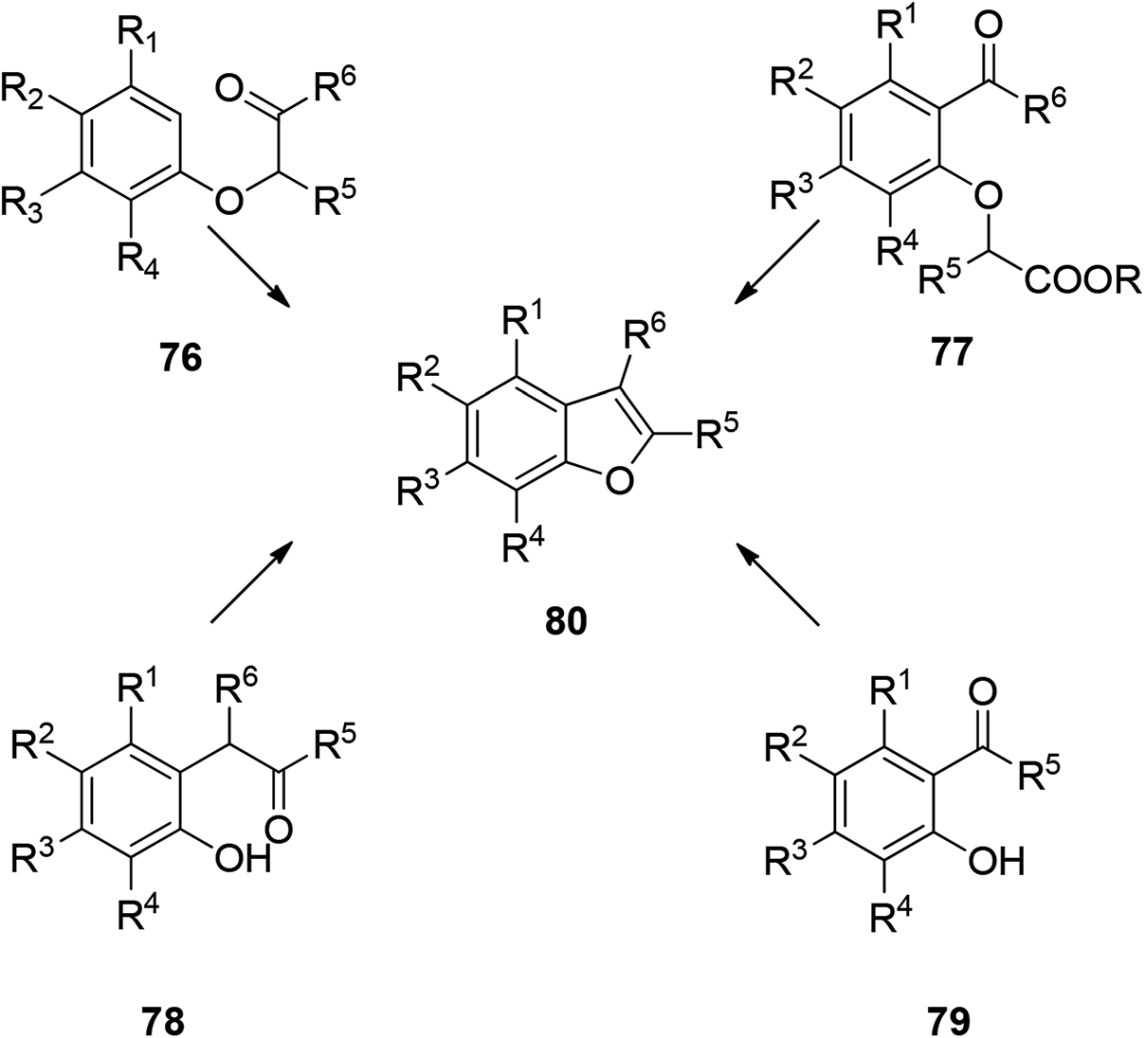
Common four methods for synthesizing benzofuran.

There are many ways to construct benzofuran rings. The most common methods are dehydration of phenoxyalkanone under acidic condition,^104^ dehydration of *o*-hydroxybenzophenone under acidic condition,^105^ decarboxylation of *o*-acetylphenoxyacetic acid or ester under alkaline condition,^106^ and cyclization of *o*-hydroxybenzophenone to construct benzofuran ring.^107^ These four types are the traditional methods of constructing benzofuran ring. In addition to the well-known typical methods for construction of benzofuran rings, there are also some specially named reactions and photochemical reaction. These abundant reaction types indicate that the construction of benzofuran ring is well developed in organic synthesis, and this provides an excellent foundation for further development of new synthetic methods in the future (Scheme 3).

### 5.1 Photochemical reaction

**Scheme 4:**
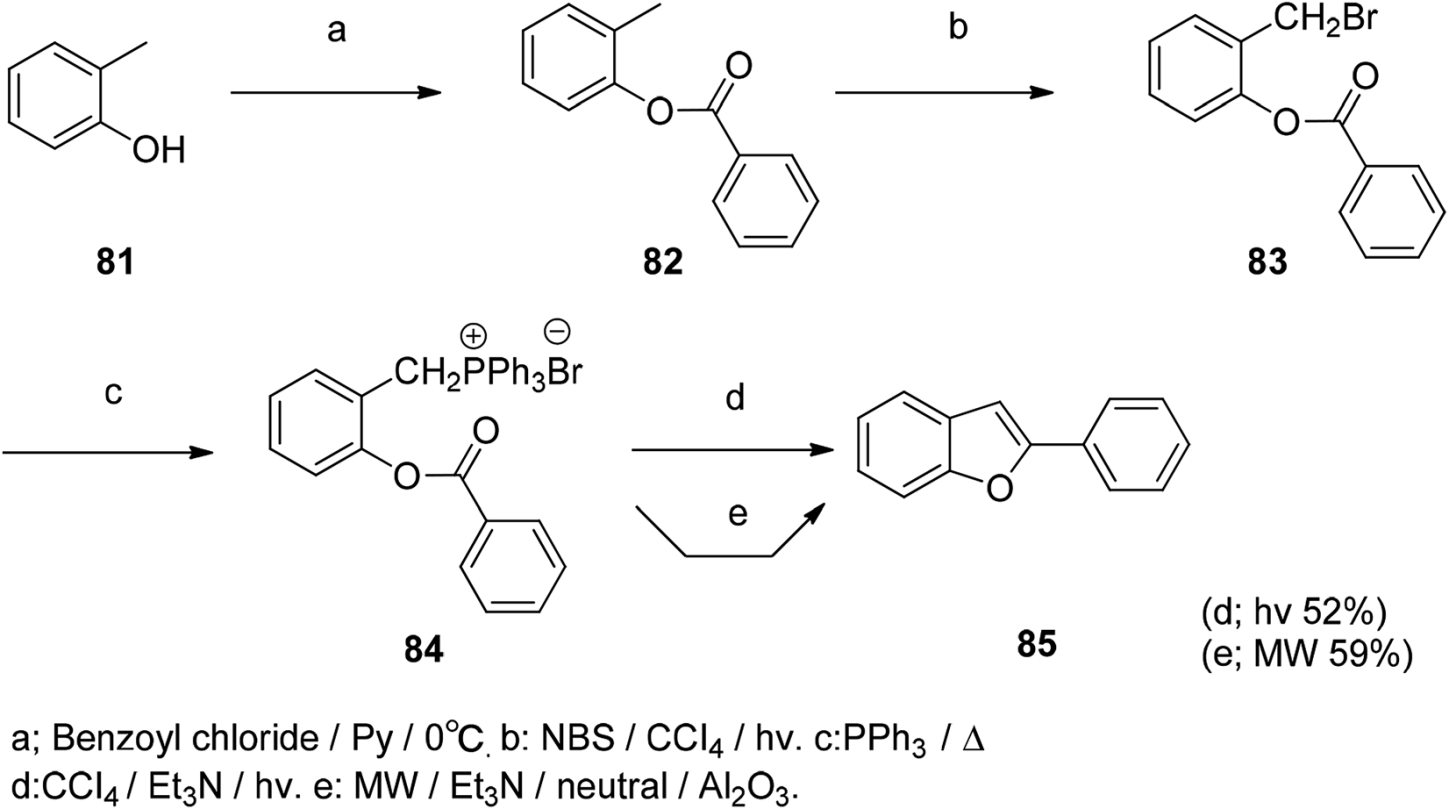
The benzofuran compound was synthesized by the photochemical reaction method under both *hv* and MW conditions.

It is well known that photochemical synthesis methods are very interesting in organic synthesis. For example, the Suzuki–Miyaura type coupling reaction uses photochemistry for organic synthesis. Scheme 4 describes a new method for the synthesis of benzofurans. The synthesis of 2-aryl/alkyl benzofurans by intramolecular photochemical Wittig reaction is achieved by photochemical reaction. There are fewer additional by-products in the reaction and can be controlled while the reaction also meets the green chemical requirements, but the compound yield is relatively low in this reaction (Scheme 4).^108^

### 5.2 Coupling reaction

#### 5.2.1 Sonogashira reaction

**Scheme 5:**
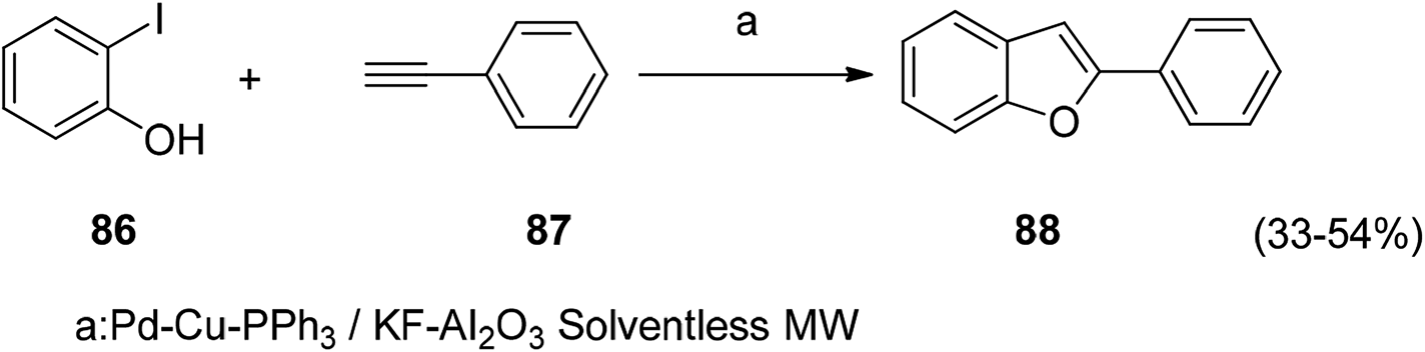
The benzofuran ring was constructed by a coupling-cyclization reaction of *o*-iodophenol with a terminal alkyne.

**Scheme 6:**
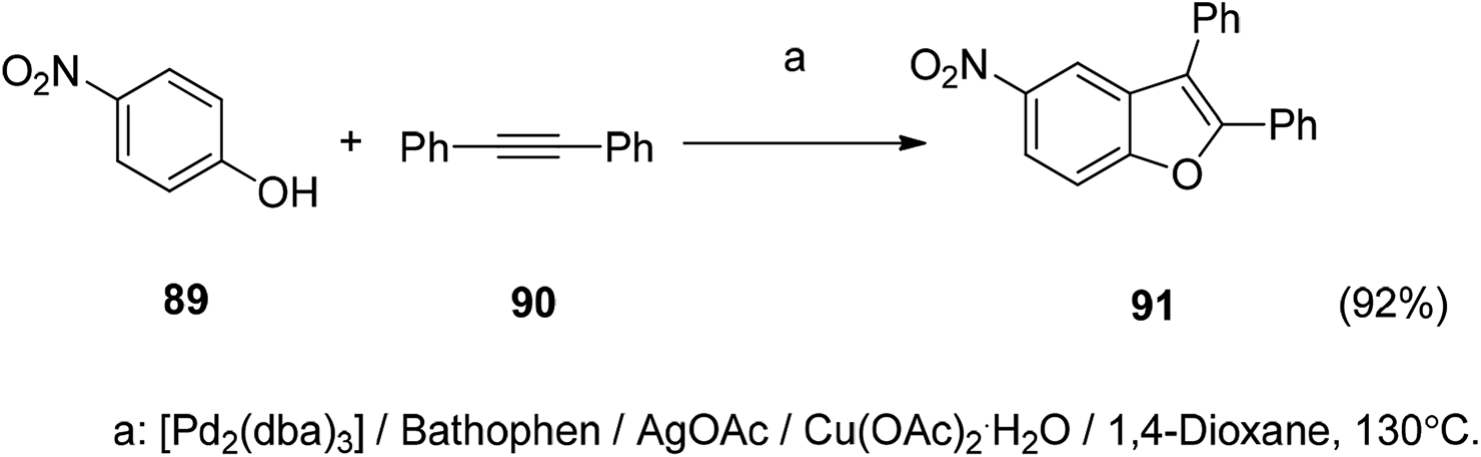
Synthesis of 2,3-diphenyl benzofuran *via* coupling of phenol with diphenylacetylene.

In the synthesis of 2,3-disubstituted benzofuran, it can be completed by a one-step reaction. It is prepared by coupling-cyclizing *o*-iodophenol with a terminal alkyne in the presence of a powder of potassium fluoride doped alumina in the presence of a mixture of palladium in the form of powder, cuprous iodide, and triphenylphosphine. The organic reaction starting materials are abundant and readily available, and most compounds have higher yields. Detailed studies on the optimization of reaction conditions and reaction mechanisms for such reactions are the subject of the future (Schemes 5 and 6).^109,110^

**Scheme 7:**
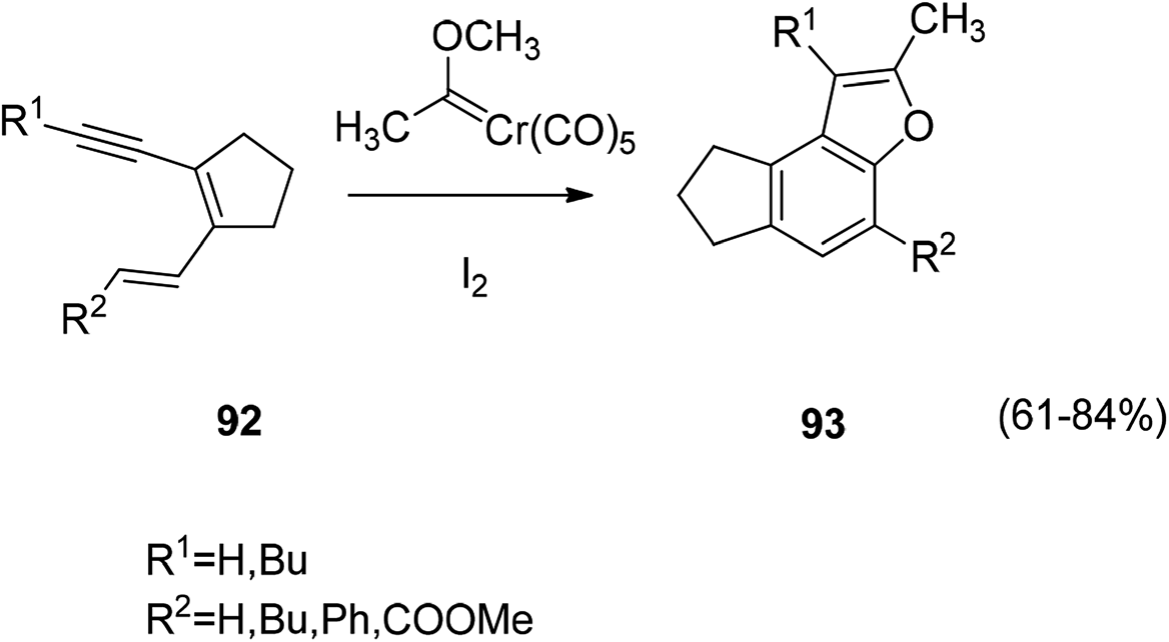
Constructed of benzofuran ring by coupling reaction.

A method of constructing a benzofuran ring by a coupling reaction of a diene acetylene compound with a Fischer carbene complex such as Scheme 7. The reaction is treated with iodine to give an uncomplexed benzofuran derivative (Scheme 7).^111^

#### 5.2.2 Sonogashira reaction (metal–organic compound)

**Scheme 8:**
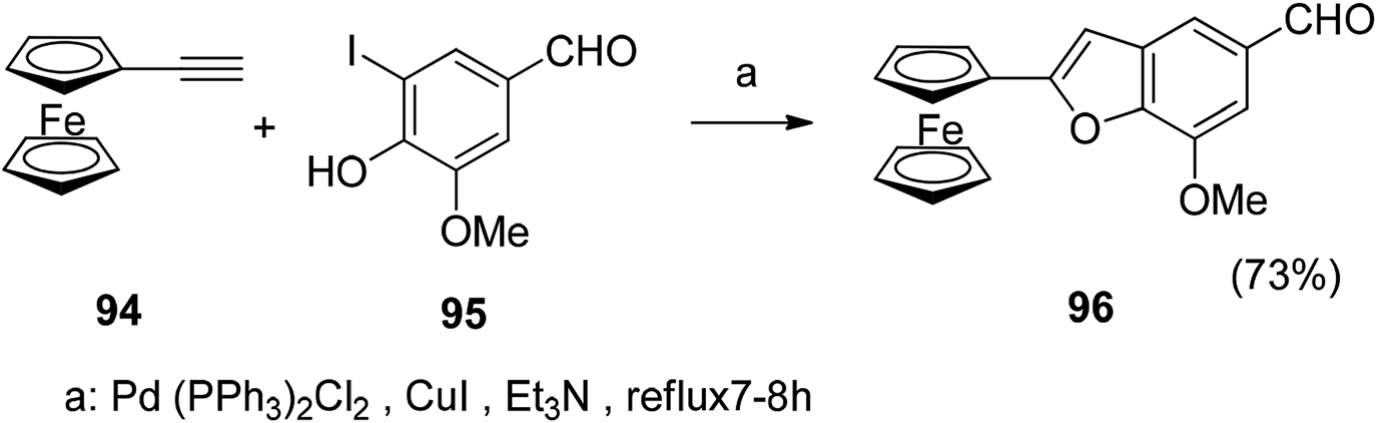
The metal-containing benzofuran 102 was prepared by a Sonogashira coupling reaction.

The Sonogashira coupling reaction of the metal–organic compound is carried out by a ring closure reaction to prepare a metal-containing benzofuran compound. Ferrocene acetylene and 5-iodovanillin are reacted in Pd (PPh_3_)_2_Cl_2_ with CuI and the yield of the metal–organic compound obtained by the reaction is high (Scheme 8).^112^

### 5.3 Knoevenegal

**Scheme 9:**
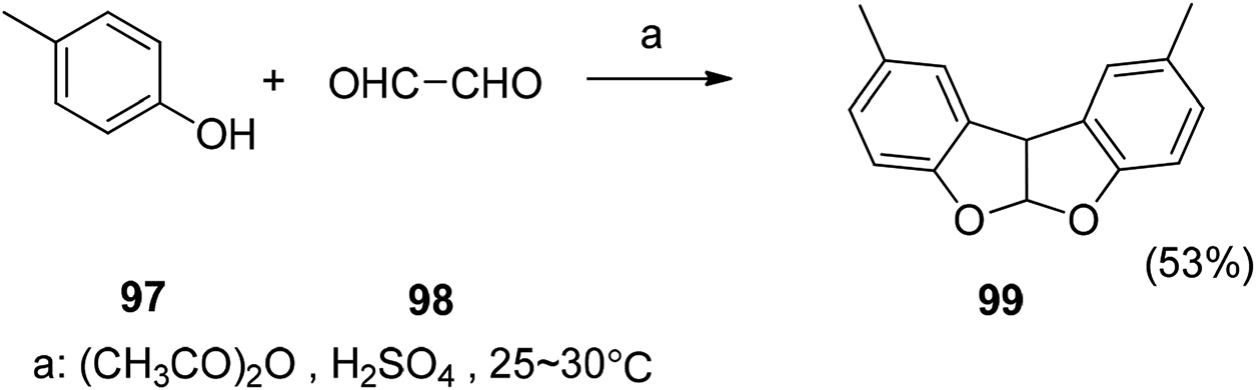
Preparation of compound 99 by Knoevenegal reaction.

Polymer II was synthesized by Knoevenagel polycondensation under the guidance of new nonlinear optics (NLO photons). The process is first carried out in anhydrous THF, followed by solid state polycondensation, and finally a backbone polymer having a high yield can be obtained. It is found that the shoulder-to-shoulder arrangement of the NLO chromophore introduced a backbone polymer with a large off-diagonal tensor component (Scheme 9).^113,114^

### 5.4 Intramolecular Heck reaction

**Scheme 10:**
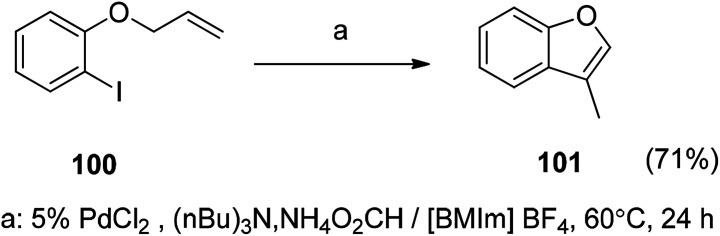
Synthesis of benzofuran in ionic liquid by a PdCl_2_-catalyzed intramolecular Heck reaction.

The Pd-catalyzed intramolecular Heck reaction in the construction of the benzofuran ring is a well-known reaction. The advantage of Pd-catalyzed intramolecular Heck reaction by ionic liquid catalysis is also evident (*e.g.*, Scheme 10). This type of ionic liquid is an ideal fixative to increase the rate and selectivity of the organic reaction, while post-treatment of the experiment is also simple. The ionic liquid with metal catalyst can be recycled and reused, which is also in line with the concept of current green chemistry.^115^

### 5.5 Heterocyclization

**Scheme 11:**
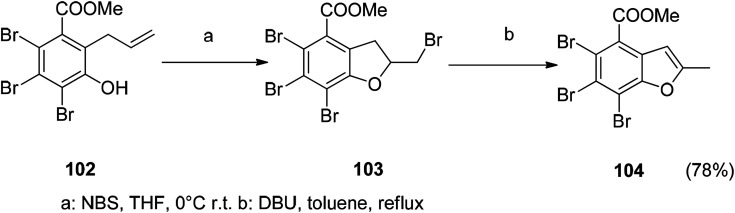
The benzofuran compound 106 was synthesized by a heterocyclization reaction.

The benzofuran derivative is obtained by NBS or NIS mediated cyclization and then post-treated with NaOMe or DBU. The overall yield of the reaction is relatively good, and the reaction conditions are mild. Therefore, the construction of a benzofuran ring by NBS or NIS-mediated electrophilic cyclization is a worthwhile approach (Scheme 11).^116^

## 6. Synthesis of various active compounds

With the popularization of the concept of green chemistry and the requirements for high yield and low toxicity of synthesis methods, the optimization and innovation of the synthetic routes of benzofuran compounds have made great progress in recent years. The construction of complex benzofuran ring using different methods also accelerated the clinical application of this kind of drugs. In this part, the synthetic methods of benzofuran compounds are illustrated with examples according to the relevant pharmacological activities of these compounds described above.

### 6.1 Synthesis of antitumor benzofuran derivatives

**Scheme 12:**
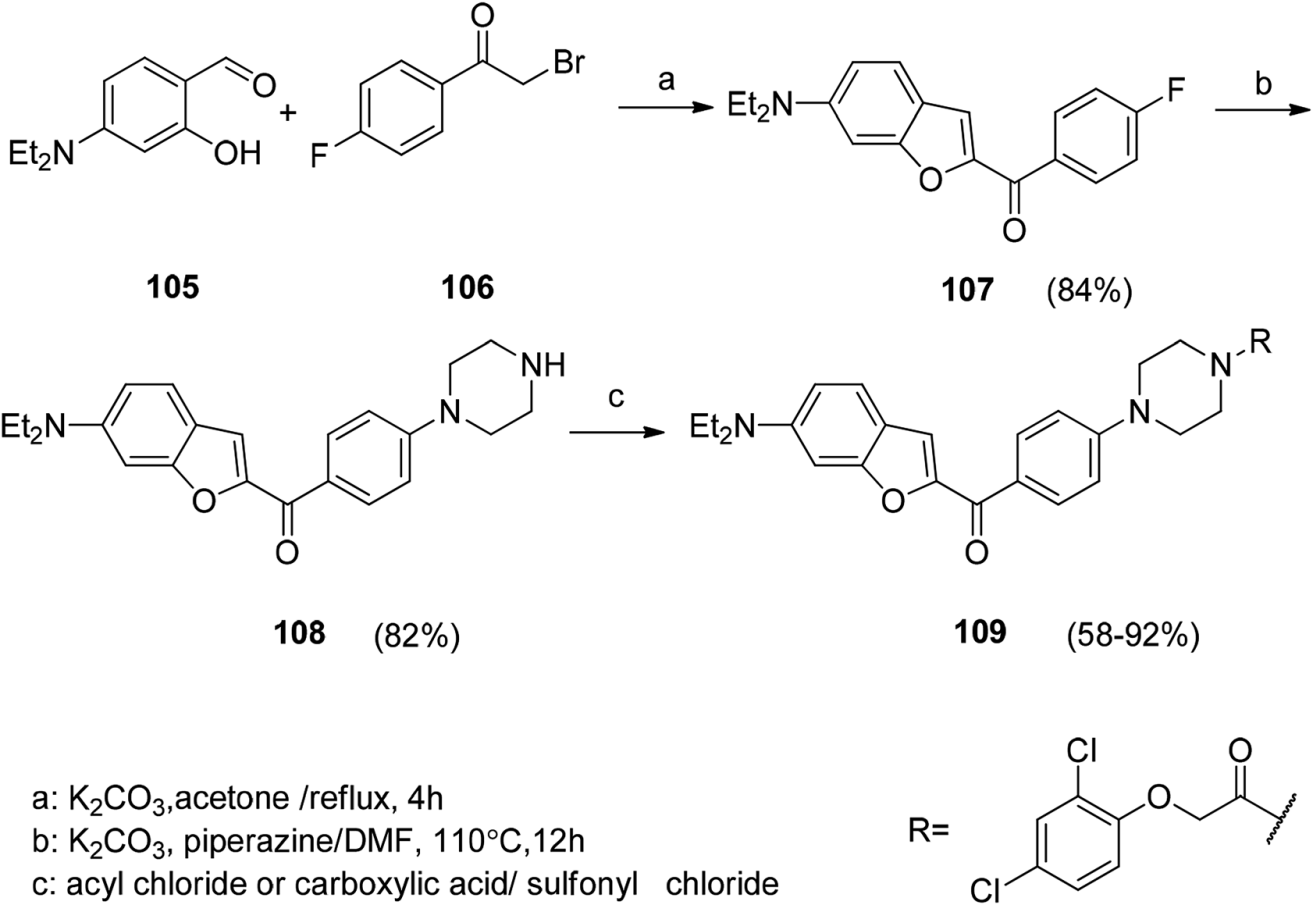
Synthetic routes of hybrid derivatives.

A series of novel hybrid compounds between benzofuran and *N*-aryl piperazine were designed and synthesized. The *in vitro* antitumor analysis results indicated that the amide derivatives generally have a higher biological activity than the sulfonamide compounds, and the chlorine or trifluoromethyl substituent is critical for regulating their cytotoxicity. The synthesis of these compounds is shown in Scheme 11. Compound 109 was found to be the most potent compound against four human tumor cell lines (Scheme 12).^117^

**Scheme 13:**
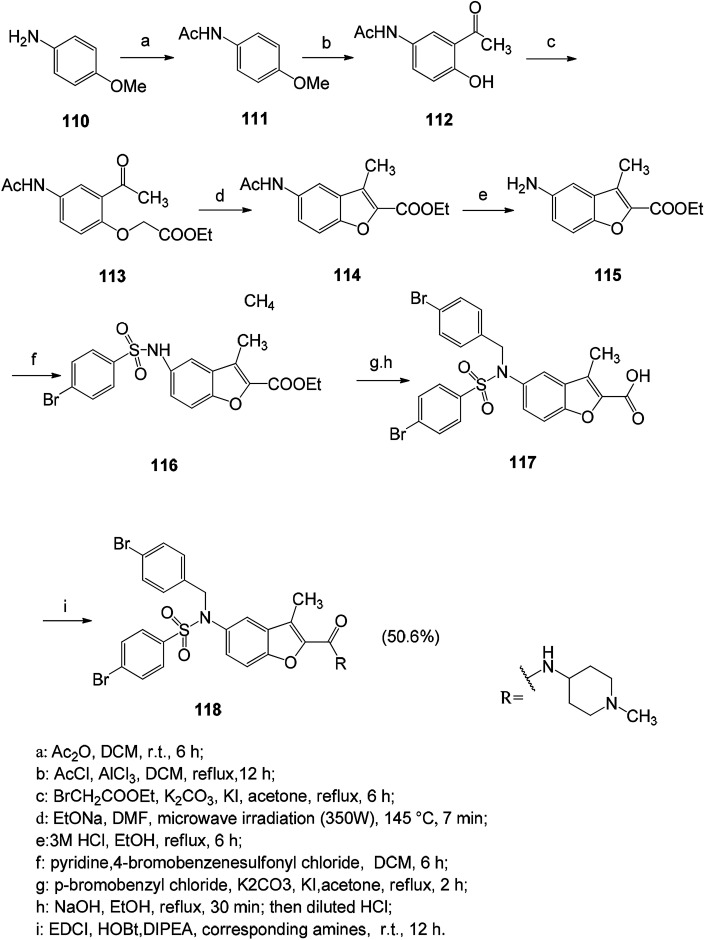
Synthetic path of compound 118.

It is a very viable strategy to design and synthesize new series of benzofuran derivatives with new scaffolds. Compounds containing heteroatoms have better physicochemical and pharmacokinetic properties than previously reported compounds. Compound 118 has a good proliferation inhibitory effect on tumor cells, and it has been found that the antitumor activity of such compounds is related to the anti-angiogenic activity *via* the anti-HIF-1α pathway (Scheme 13).^9^

**Scheme 14:**
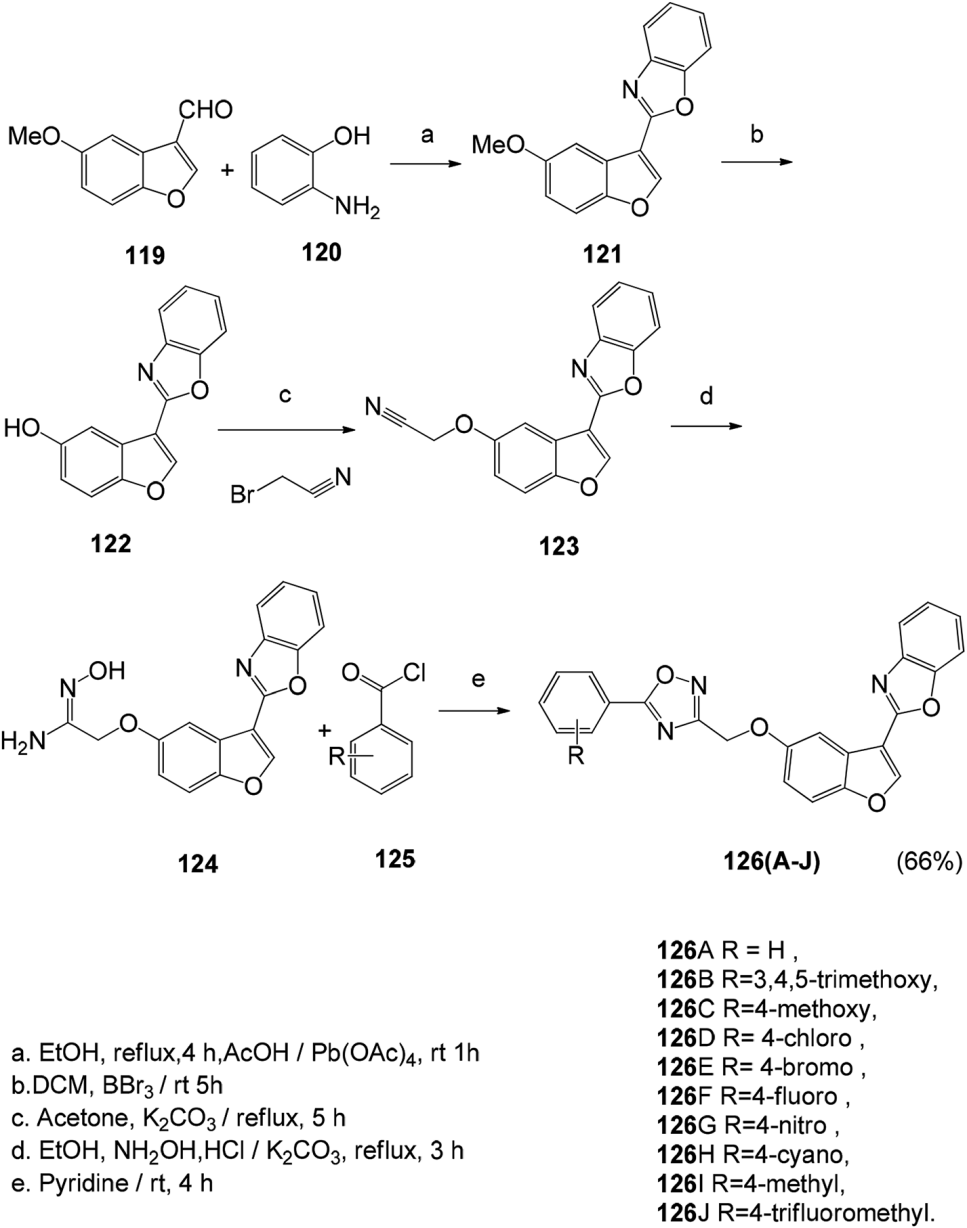
Synthesis route of 1,2,4-oxadiazole fused benzofuran derivatives (126A–126J).

The novel 1,2,4-oxadiazole fused benzofuran derivatives (126A–126J) were synthesized. The anticancer properties of these compounds were determined by MTT methods using a series of cancer cell lines. Combretastatin-A4 was used as a positive control, in which compounds 126B, 126C, 126D, 126G, 126H and 126J showed better activity than the positive control (Scheme 14).^118^

### 6.2 Synthesis of anti-bacterial benzofuran derivatives

Synthetic route of benzofuran compound 53 with antibacterial activity.

**Scheme 15:**
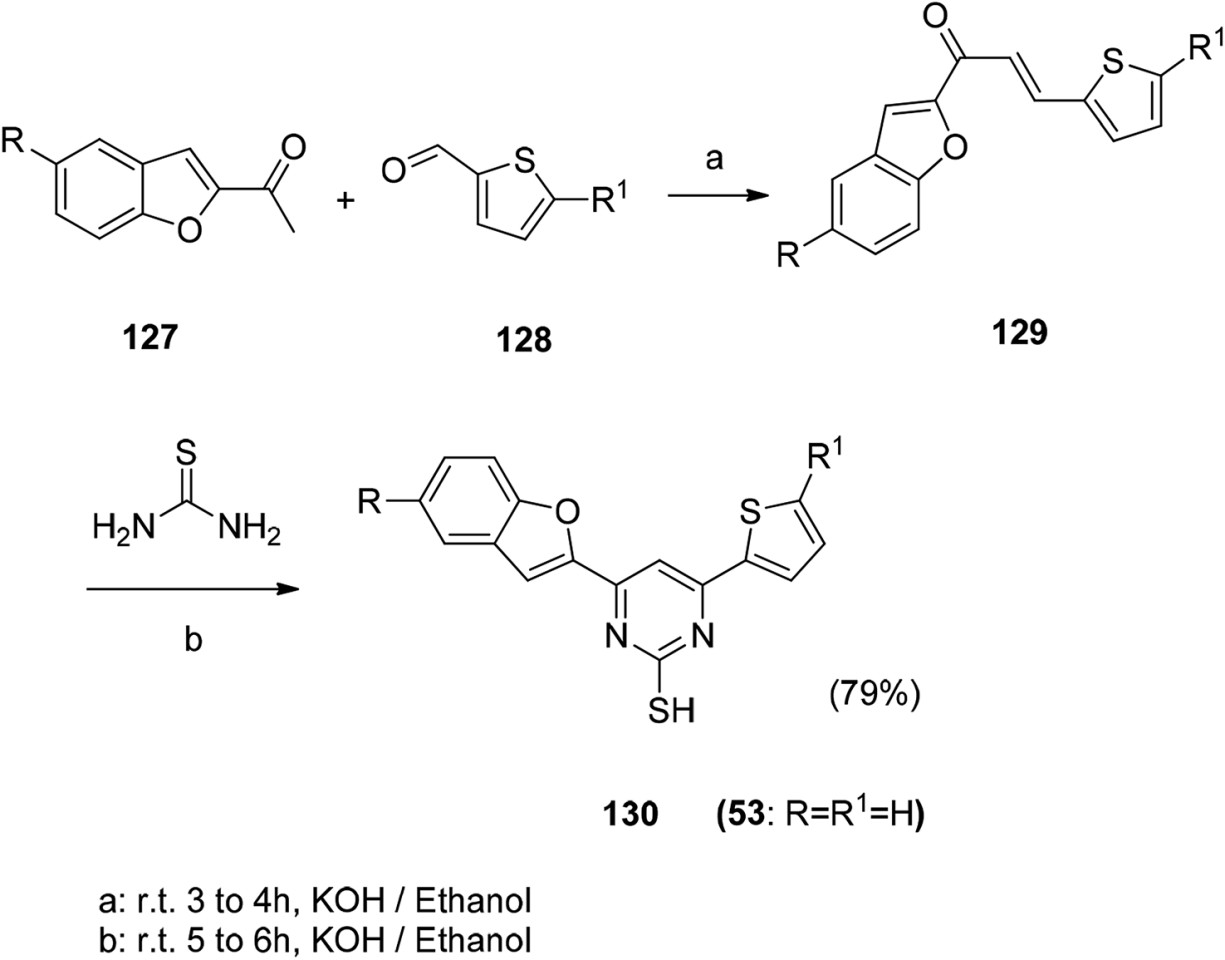
Synthesis of benzofuran derivative 130. Reagents and conditions.

**Scheme 16:**
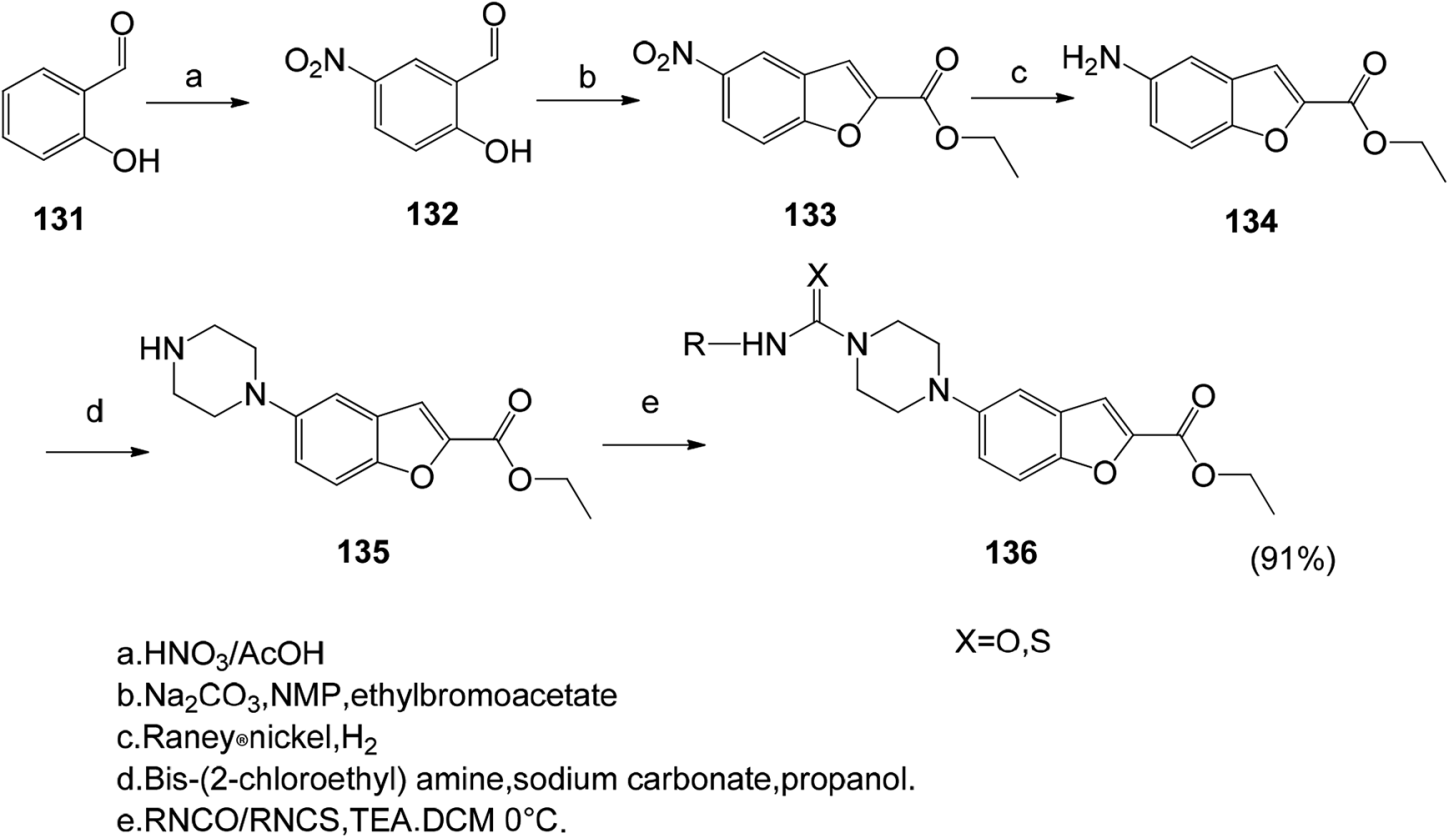
Synthesis of ethyl 5-aminobenzofuran-2-carboxylate compound 136.

The benzofuran based lead compounds were synthesized, and the obtained 2-hydroxy-5-nitrobenzaldehyde (132) was subsequently treated with ethyl bromoacetate in the presence of sodium carbonate in *N*-methyl pyrrolidine, and the ethyl 5-nitrobenzofuran-2-carboxylate was obtained in good yields and purity.^119^ Subsequent reduction of the nitro group at the 5th position of the benzofuran ring gives the corresponding ethyl 5-aminobenzofuran-2-carboxylate (136) compound. The advantage of this method is the high synthesis yield (Schemes 15 and 16).

**Scheme 17:**
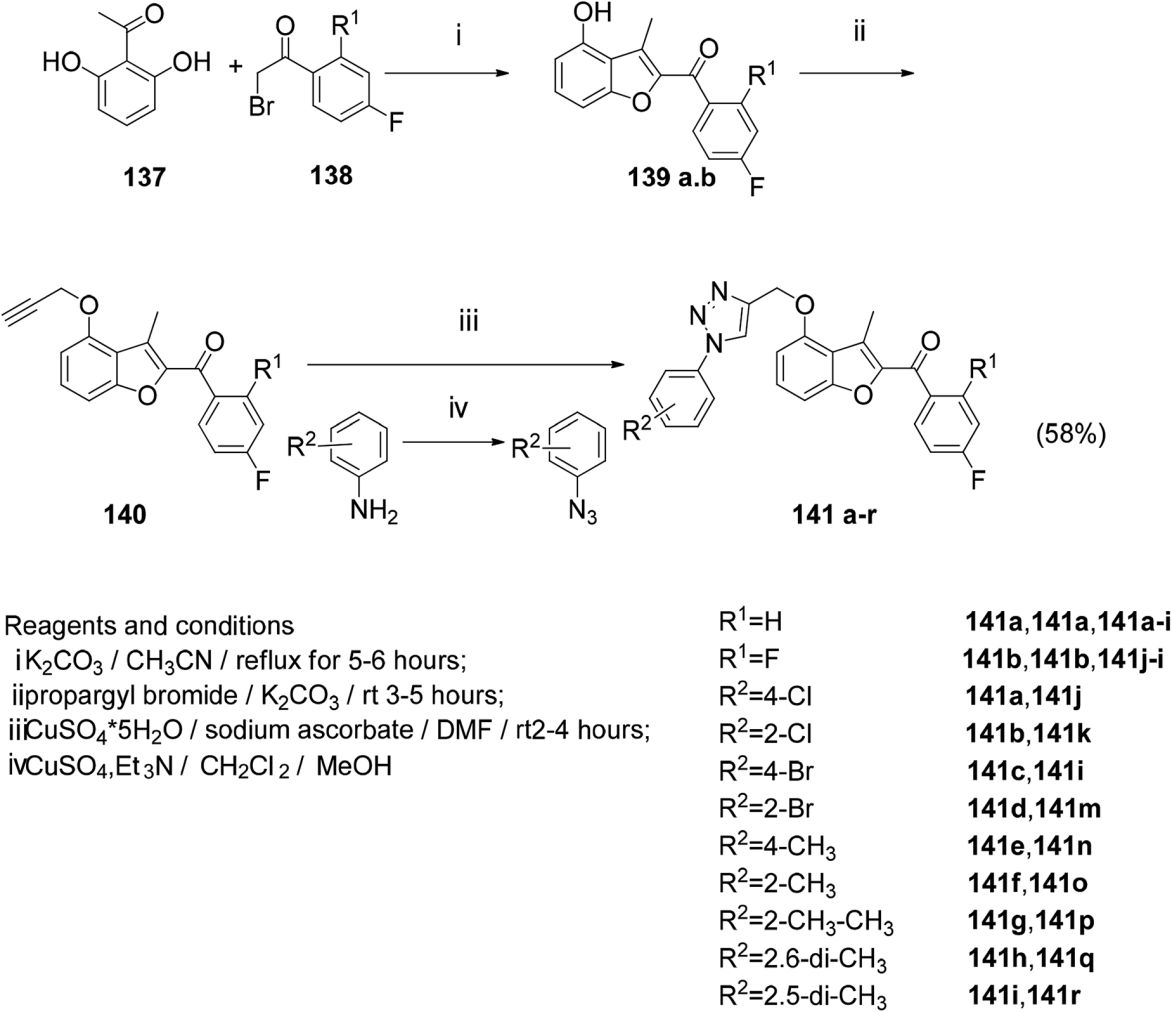
Synthesis of the target compound 141a–r.

A series of novel benzofuran-triazole hybrids were designed by click chemistry and found to have moderate to satisfactory antifungal activity by testing the target compounds.^10^ The reaction of 2′,6′-dihydroxyacetophenone with the corresponding 2-bromoacetophenone under the modified Rap–Stoermer reaction condition gave the hydroxy alkylation of the benzofuran scaffold (139a, b) and the propargyl bromide compound to obtain the end Alkyne derivative (140a, b). The aromatic azide (140a–i) was prepared from the corresponding aniline under Sandmeyer conditions. Finally, the target compound (141a–r) was obtained by click chemistry, and the yield was good (Scheme 17).^10^

**Scheme 18:**
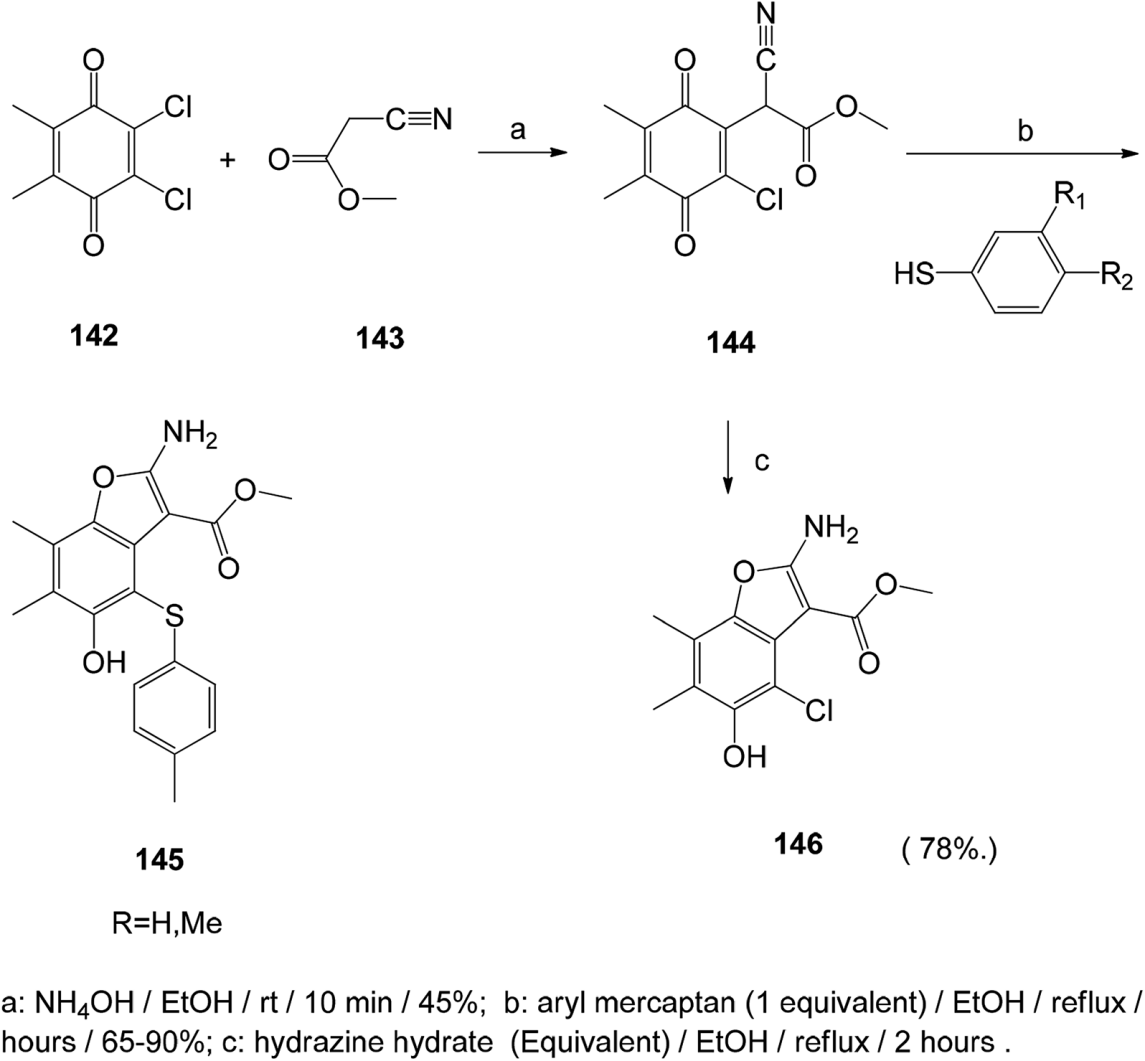
Synthesis of benzofuran-5-ol derivatives. Reagents and conditions.

The benzofuran-5-ol derivative was synthesized and tested for its *in vitro* antifungal activity against *Candida*, *Aspergillus* species and *Cryptococcus neoformans*, and it showed good antifungal activity, suggesting that benzofuran-5-ol is a promising antifungal agent (Scheme 18).^120^

**Scheme 19:**
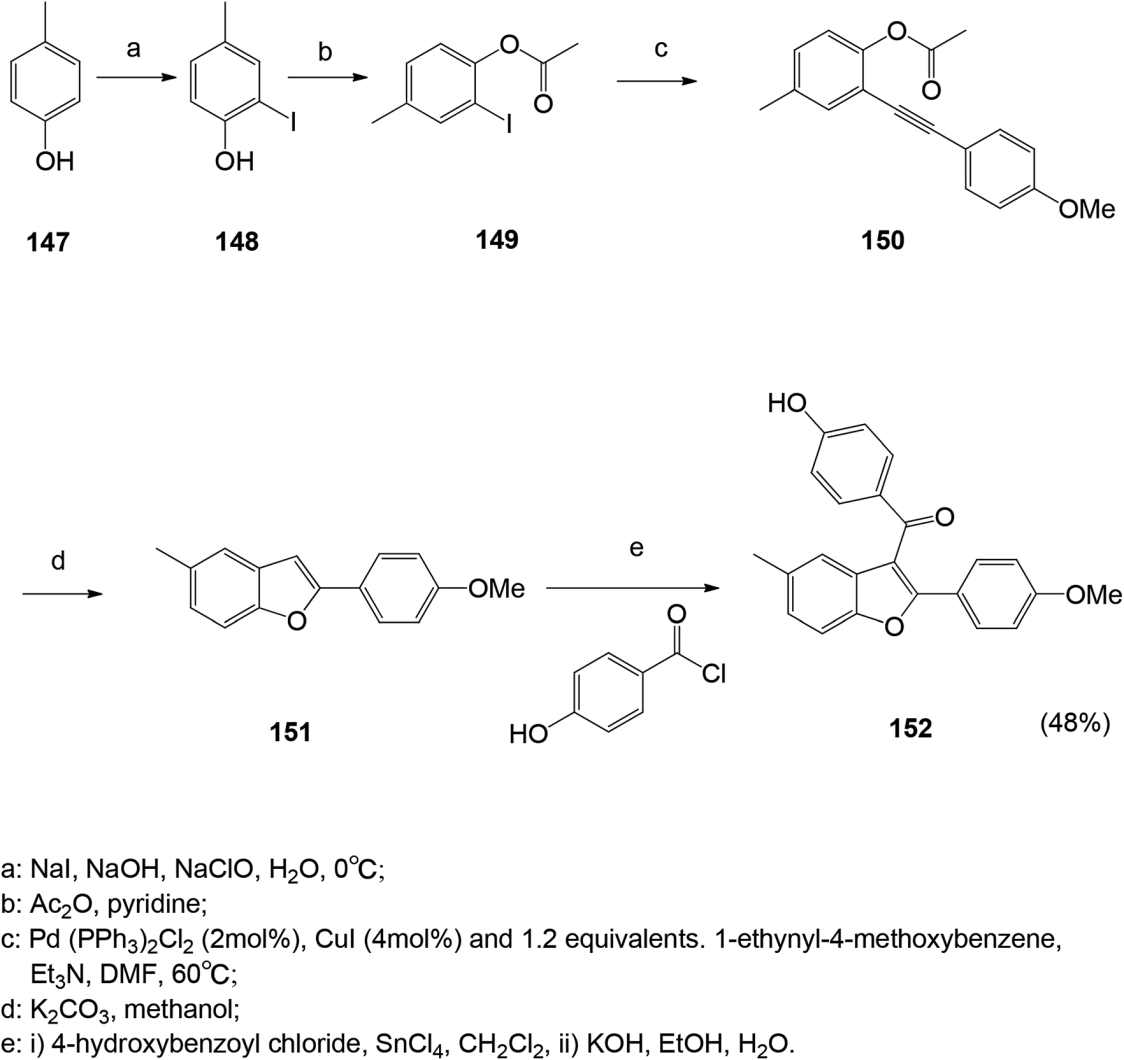
Synthesis route of benzofuran compound 152.

A series of antibacterial benzofuran compounds were designed and synthesized.^121^ The ketone ligands were synthesized at the C-3 position and their antibacterial and antifungal activities were screened. They have good biological activity against four bacterial strains *Escherichia coli*, *Staphylococcus aureus*, methicillin-resistant *Staphylococcus aureus*, *Bacillus subtilis* and fungus *Candida albicans*.^121^ Among them, compound 152 showed excellent antibacterial activity against *Staphylococcus aureus* and MRSA (Scheme 19).

Monoiodo-phenol was obtained by iodinating phenol with NaI in the presence of NaOH and NaClO at low temperature (0 °C) and treated with acetyl chloride to obtain an intermediate. The combination of palladium and cuprous catalysts is typically used for the cross-coupling of aryl halides with terminal acetylenes to yield the corresponding aryl acetylenes.^122^ Intramolecular cyclization of the compound stilbene in potassium by Friedel–Crafts method, treatment of commercially available benzoic acid with SOCl_2_, followed by the addition of tin chloride to obtain the final compound. The acetylated compounds are hydrolyzed with KOH to give their respective phenolic compounds.^121^ In palladium-catalyzed reactions, alkyne substrates have been widely used to form carbon–carbon bonds, resulting in cyclic and polycyclic structures of macrocyclic isoflavones and chromene quinoline derivatives γ-butyrolactone and hydrazine. Therefore, the method for synthesizing benzofuran from *o*-iodophenol and acetylenic compounds under palladium catalysis is convenient and versatile.^123,124^

### 6.3 Synthesis of antiviral benzofuran compounds

**Scheme 20:**
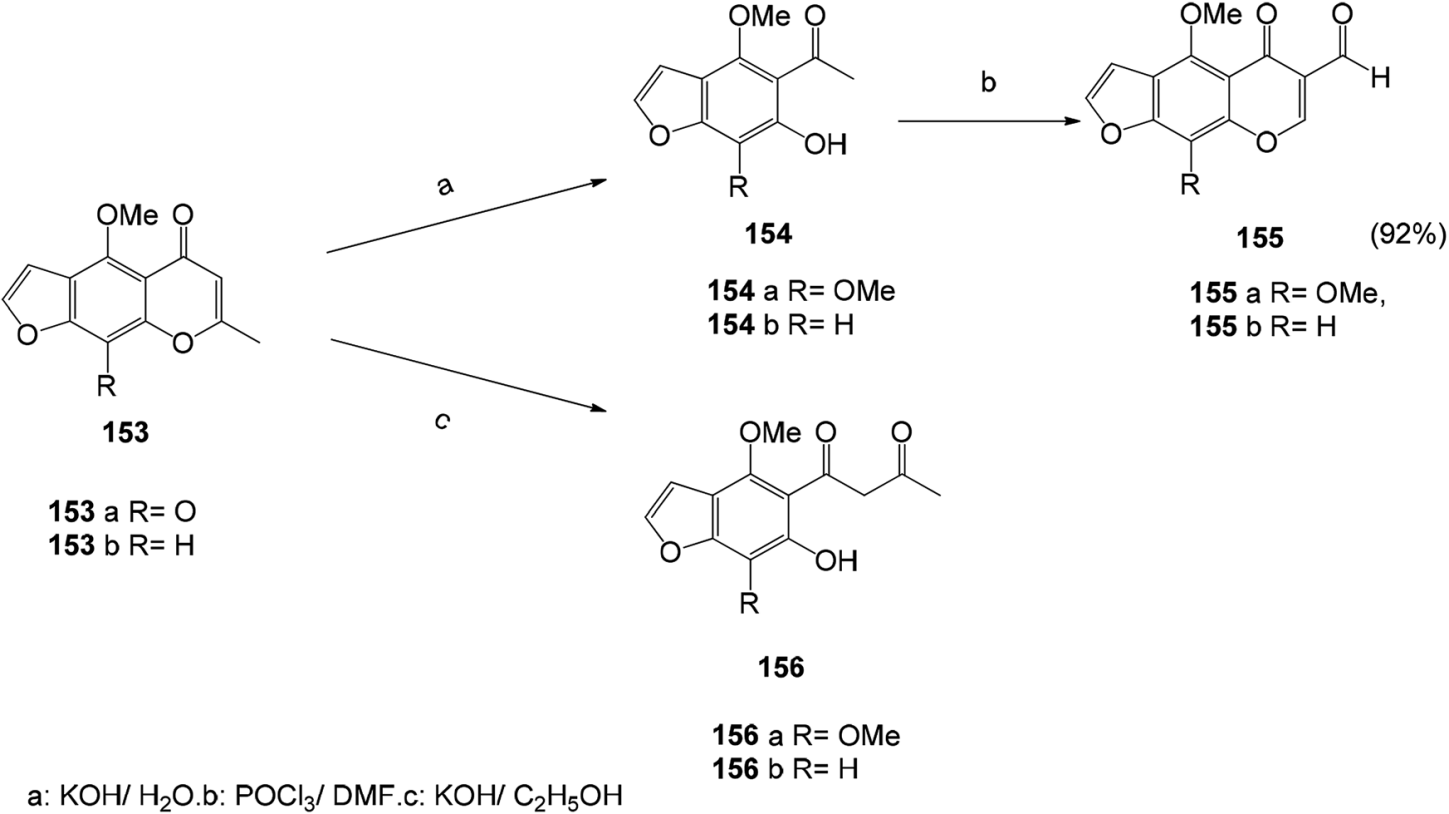
The synthesis route of 5-oxo-5*H*-furo[3,2-*g*]chromene-6-carboxaldehyde by the Vilsmeier–Haack reaction starting from compounds 154a and 154b.

**Scheme 21:**
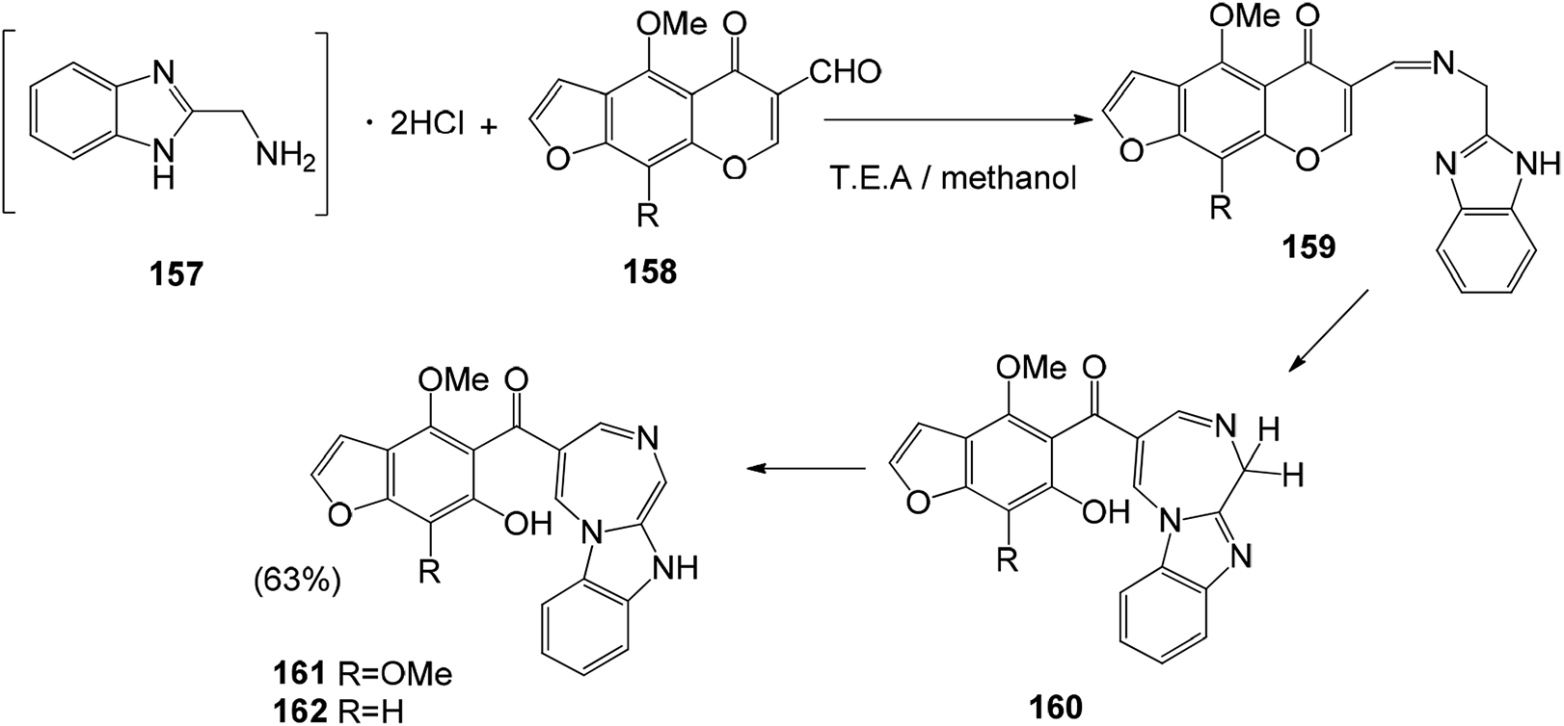
Synthetic route of compounds 161 and 162.

A series of new potent antiviral benzofuran derivatives can be synthesized by naturally occurring furanone compounds (khellin (153a) and visnagin (153b)). Khellin (153a) and visnagin (153b) are very sensitive to alkali. The solvent used in the reaction has a great influence on the obtained product. Alcohol hydrolysis of 153a and 153b with potassium hydroxide gives different products, namely x-acetyl ketone (156a) and x-acetylisoxanthone (156b). The hydrolysate is an important molecule for the synthesis of new furanone. Compounds 154a and 154b were directly used in the synthesis of 5-oxo-5*H*-furo[3,2-g] chromene-6-formaldehyde by Vilsmeier–Haack reaction^125^ (Schemes 20 and 21).

### 6.4 Synthesis of antioxidant active benzofuran derivatives

**Scheme 22:**
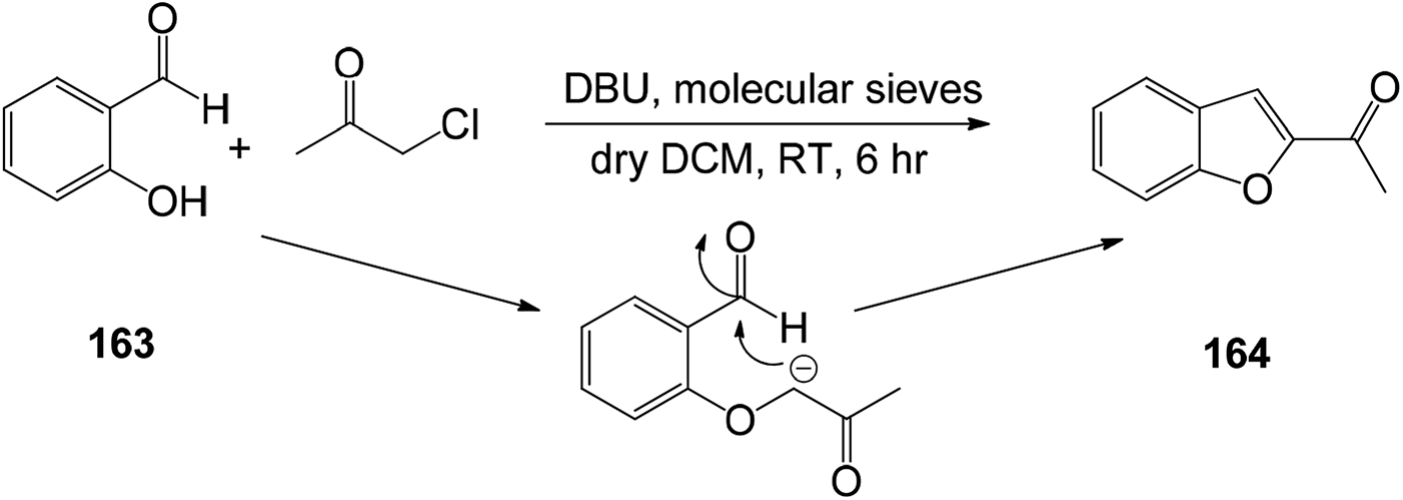
Synthesis of 2-acetyl benzofuran 164.

**Scheme 23:**
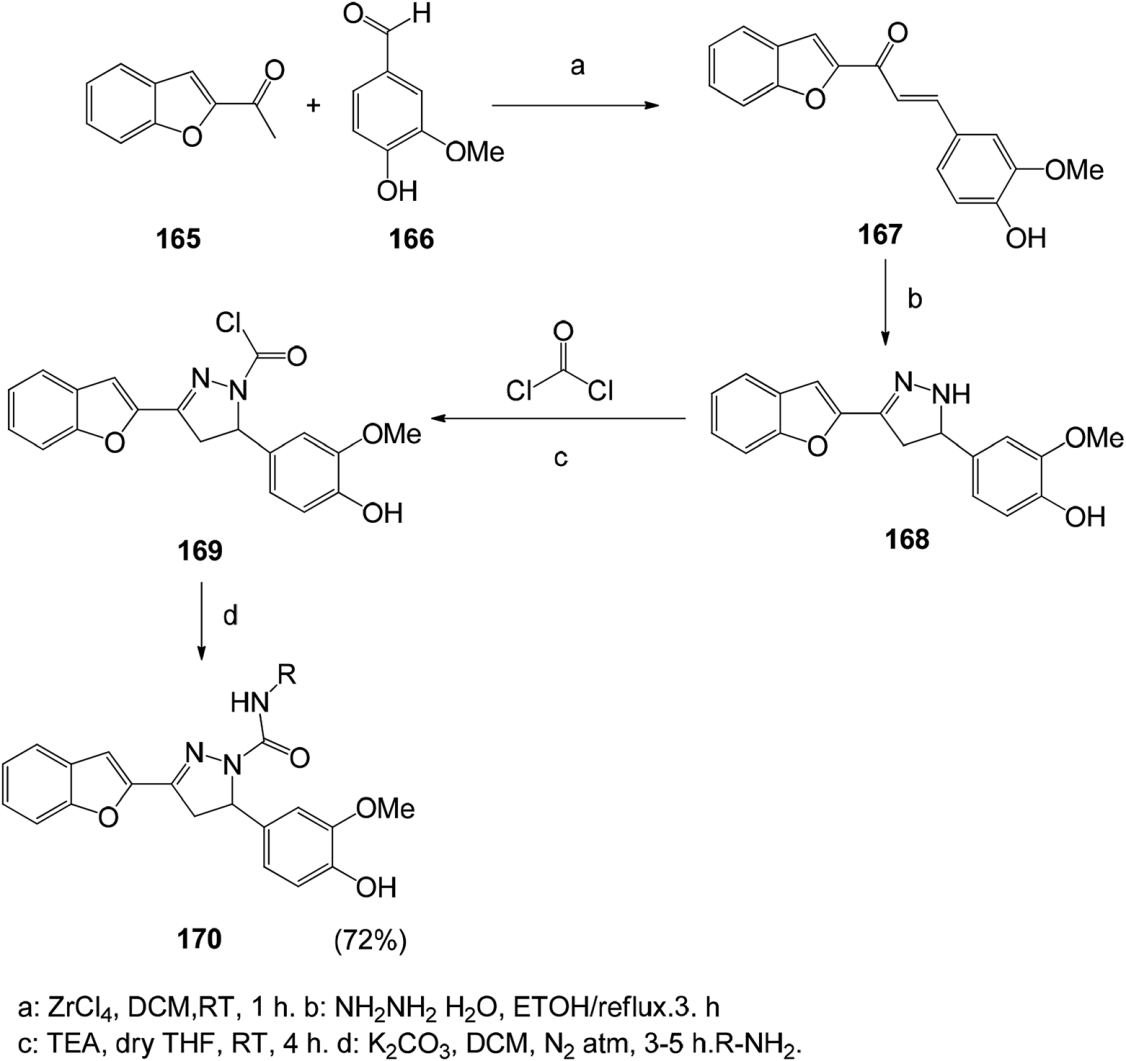
Reaction pathway for the synthesis of benzofuran based 1,3,5-substituted pyrazole derivatives 170.

In order to find new antioxidants and antibacterial agents with improved efficacy, a series of benzofuranyl 1,3,5-substituted pyrazole analogs were synthesized (Schemes 22 and 23). Screening for their antioxidant activity indicated that these compounds have good antioxidant activity, and the synthetic route of this benzofuranyl 1,3,5-substituted pyrazole analog is as follows.^126^

**Scheme 24:**
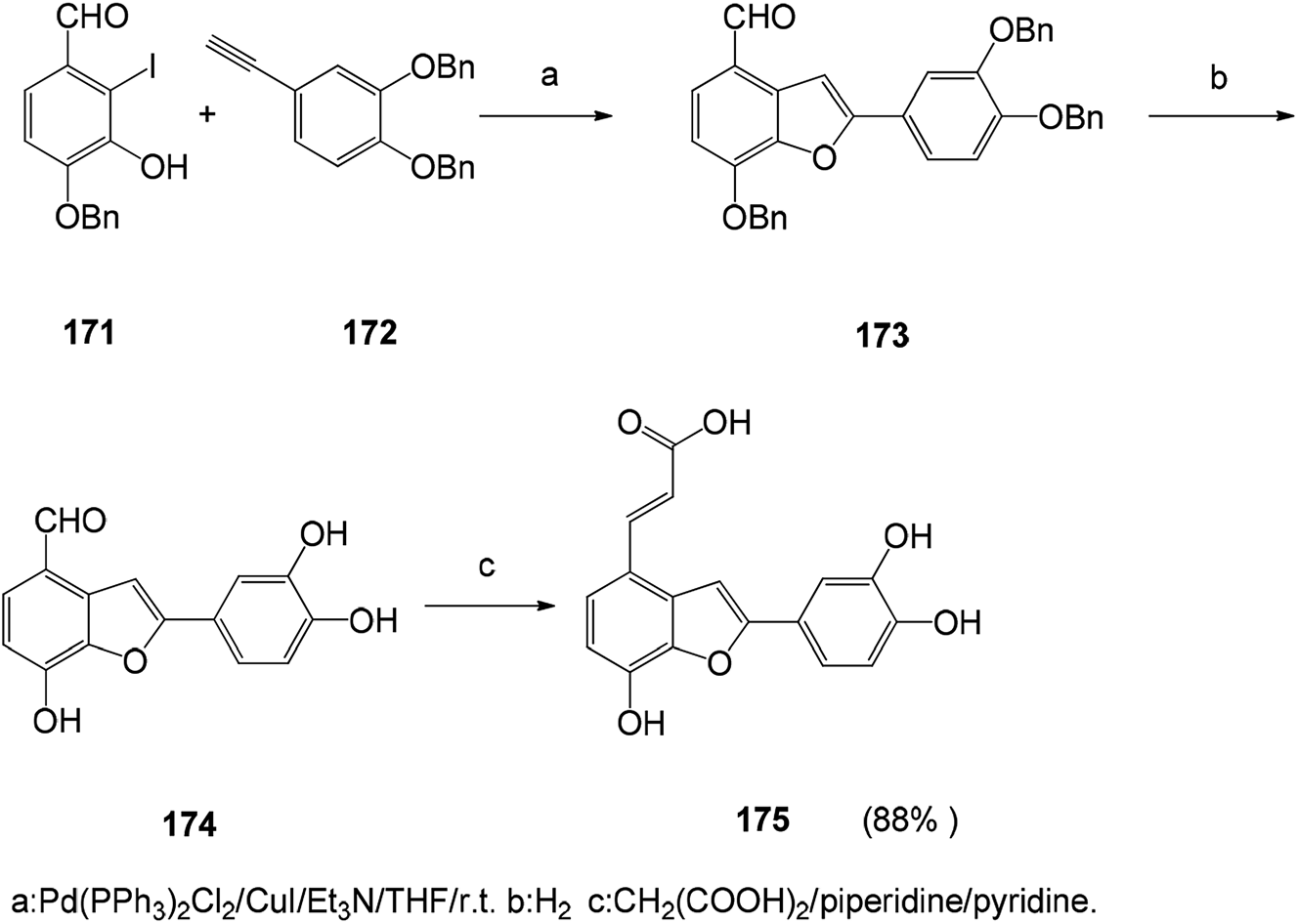
Synthetic route to salvianolic acid derivatives.

With the development of medicinal chemistry, palladium-mediated synthesis of common synthons and antioxidant analogs for the synthesis of naturally occurring salvianolic acid has been proposed (Scheme 24). Synthetic pathways can be used to obtain analogs with balanced lipophilic/hydrophilic properties that can lead to potentially interesting LDL antioxidants for prevention of cardiovascular disease.^127^ The 2-substituted benzofuran compound is synthesized by heterocyclic reaction of *o*-iodophenol with an acetylene substrate containing a terminal acetylene group. This synthetic route has focused on the use of palladium catalysts to form carbon–carbon bonds and carbon-heteroatom bonds. This route is also a versatile and simple method for the synthesis of benzofuran compounds.^128–130^

**Scheme 25:**
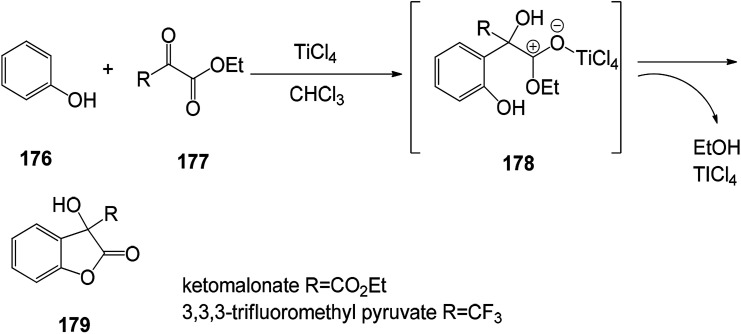
Friedel–Crafts alkylation/lactonization of polyphenols was carried out using TiCl_4_ as a catalyst.

**Scheme 26:**
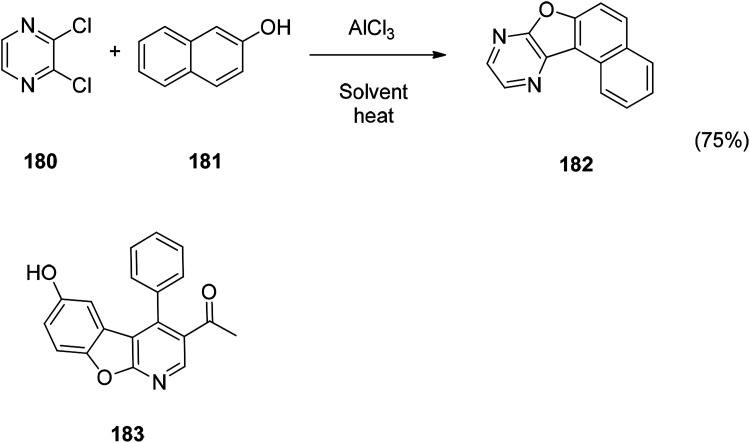
Benzofuran fused compound structure.

The benzofuran-2-one derivatives were synthesized (Scheme 25) and found to have good antioxidant activity. The antioxidant capacity of the most stable compounds was evaluated by both DPPH assay and cyclic voltammetry analyses performed in alcoholic media (methanol) as well as in aprotic solvent (acetonitrile). The experimental method is a short-term and practical reaction of 3-hydroxy-3*H*-benzofuran-2-one, and the possibility of obtaining various phenol derivatives by the domino reaction involving the first Friedel-alkylation and subsequent intramolecular lactonization. Specifically, phenolic malonate or 3,3,3-trifluoromethylacetone as an electrophilic counterpart in the presence of polyphenol as a substrate and TiCl_4_ (10 mol%) as a catalyst, the acid ester 3,3,3-trifluoromethylacetone undergoes a reaction that activate the alkylation reaction (Scheme 26).^131^

## 7. Benzofuran fused derivative

The benzofuran fused derivative are also an important class of benzofuran derivatives such as usnic acid. In recent years, great progress has been made in the study of benzofuran fused derivatives. Since this strategy is believed to have the potential to increase the potency of the compound. A new one-pot synthesis of a benzofuran-fused N-heterocycle is achieved by AlCl_3_-mediated C–C followed by a C–O bond between 2,3-dichloropyrazine or a derivative thereof and phenol. The new compound can be used as a potential inhibitor of PDE4B. The docking of the compound 180 with the PDE4B protein showed that the key role was achieved *via* the inhibition of the benzofuran moiety in PDE4B. Compound 180 may also have therapeutic potential because the intended inhibitors are beneficial for the treatment of inflammatory and immune diseases.^132^ The biological evaluation of various cyclin-dependent kinase inhibitors revealed that benzofuran fused derivative 181 has a certain activity and is expected to be further developed in future studies.^133^

## 8. Conclusions and perspective

This review has highlighted the various aspects of benzofuran derivatives including their important natural product sources, biological activities and drug prospects, and chemical synthesis. Benzofuran compounds exhibit potent biological properties including analgesic, anti-inflammatory, antibacterial, antifungal, antitumor, antiviral, and enzyme inhibitory activities. There are also fluorescently active benzofuran compounds which have received increasing attention. Substitutions at the C-2, C-3 position in the benzofuran ring, as well as compounds substituted on the phenyl ring, encompass most of the benzofuran derivative ring systems. The most well-known benzofuran derivatives are amiodarone, geranium xanthine toxin, bergapten, globulin and usnic acid compounds, most of which have been used as lead compounds in drug design and new drug development.

Benzofuran scaffolds have a wide range of biological activities, and the review of such compounds can further understand the application of such compounds in medicinal chemistry. This review details the various pharmacological activities of benzofuran derivatives and several compounds that have been successfully used in clinical practice. The clinical pharmacological activities of these compounds provide a new basis for the study of benzofuran analogs and novel potential scaffolds. In the synthesis of benzofuran compounds, this review summarizes the synthesis of benzofurans by classifying the activity of the compounds. These methods can be further used to synthesize benzofuran compounds with promising active structural units. In the natural source part, by summarizing the activity and structure of the natural products of benzofuran in the past ten years, important references are provided for structural modification of natural products in the field of medicinal chemistry to improve biological activity. Although great progresses have been made in benzofuran skeleton activity and synthesis research the structure optimization and modification of benzofuran compounds still needs more work to improve the selectivity of the compounds. It is hoped that the ideas in this review article and the cited examples will motivate and further optimize the full potential of benzofuran compounds, to improve the design selectivity, optimization and multifunctional opportunities of benzofuran compounds, and to help in the treatment of multifactorial diseases in the future.

## Conflicts of interest

The authors declare that this article content has no conflict of interest.

## Supplementary Material
